# Intra-brain vascular models within the ICRP mesh-type adult reference phantoms for applications to internal dosimetry

**DOI:** 10.1088/1361-6560/acc926

**Published:** 2023-05-02

**Authors:** Camilo M Correa-Alfonso, Julia D Withrow, Sean J Domal, Bonnie N President, Robert J Dawson, Lucas McCullum, Chris Beekman, Clemens Grassberger, Harald Paganetti, Wesley E Bolch

**Affiliations:** 1 Medical Physics Program, College of Medicine, University of Florida, Gainesville, FL 32611 United States of America; 2 J. Crayton Pruitt Family Dept. of Biomedical Eng., University of Florida, Gainesville, FL 32611 United States of America; 3 Massachusetts General Hospital, Harvard Medical School, Boston, MA 02115 United States of America

**Keywords:** brain, blood vasculature, ICRP reference phantoms, radionuclide *S* values, radiopharmaceutical dosimetry

## Abstract

**Objective.:**

Phantoms of the International Commission on Radiological Protection provide a framework for standardized dosimetry. The modeling of internal blood vessels—essential to tracking circulating blood cells exposed during external beam radiotherapy and to account for radiopharmaceutical decays while still in blood circulation—is, however, limited to the major inter-organ arteries and veins. Intra-organ blood is accounted for only through the assignment of a homogeneous mixture of parenchyma and blood [single-region (SR) organs]. Our goal was to develop explicit dual-region (DR) models of intra-organ blood vasculature of the adult male brain (AMB) and adult female brain (AFB).

**Approach.:**

A total of 4000 vessels were created amongst 26 vascular trees. The AMB and AFB models were then tetrahedralized for coupling to the PHITS radiation transport code. Absorbed fractions were computed for monoenergetic alpha particles, electrons, positrons, and photons for both decay sites within the blood vessels and for tissues outside these vessels. Radionuclide *S*-values were computed for 22 and 10 radionuclides commonly employed in radiopharmaceutical therapy and nuclear medicine diagnostic imaging, respectively.

**Main results.:**

For radionuclide decays, values of *S* (brain tissue ← brain blood) assessed in the traditional manner (SR) were higher than those computed using our DR models by factors of 1.92, 1.49, and 1.57 for therapeutic alpha-emitters, beta-emitters, and Auger electron-emitters, respectively in the AFB and by factors of 1.65, 1.37, and 1.42 for these same radionuclide categories in the AMB. Corresponding ratios of SR and DR values of *S*(brain tissue ← brain blood) were 1.34 (AFB) and 1.26 (AMB) for four SPECT radionuclides, and were 1.32 (AFB) and 1.24 (AMB) for six common PET radionuclides.

**Significance.:**

The methodology employed in this study can be explored in other organs of the body for proper accounting of blood self-dose for that fraction of the radiopharmaceutical still in general circulation.

## 1. Introduction

Computed tomography, magnetic resonance imaging, and ultrasonography can provide realistic 3D models of the human cerebral vasculature, with recent advances allowing the separation of the arterial and venous networks (Nowinski et al 2009). Different medical images combined with novel automatic image processing techniques have been used to obtain human-specific brain vascular models (Hsu et al 2015). Nevertheless, models based on medical imaging techniques are typically limited by spatial resolution. While recent studies using high-resolution microCT have demonstrated the ability to reconstruct the complete vascular network of the entire brain at the capillary level in adult mice via corrosion casting procedures (Quintana et al 2019), such studies are not translatable to human studies. Consequently, numerical models have been applied to develop virtual vascular models in several human organs including the brain. Vascular models based on the constrained constructive optimization (CCO) method have been reported for cerebral microcirculation (Linninger et al 2013) and in the brain cortex (Bui et al 2010). A hybrid approach that combines CT image-based geometries for the main vessels and a mathematical algorithm for the development of the mesoscale vasculature (Ii et al 2020) has been applied to the reconstruction of the superficial cortical vessels. Although these models provide detailed descriptions of the vascular pathways, many are restricted to a specific brain sub-region and none have been mainly focused on external beam or radiopharmetical dosimetry applications.

The aim of this work was to apply the previously described vessel generation algorithm by Correa-Alfonso et al (2022) to develop vascular models within the brain mesh-surface of the adult male and adult female ICRP reference phantoms (ICRP 2020) with applications for improved dose assessment for internal emitters. The adult brain mesh surface presented in ICRP Publication 145 accommodates the total volume of both brain parenchyma and its blood content—a feature absent in the ICRP Publication 110 voxel reference phantoms (ICRP 2009). Nevertheless, blood is homogeneously distributed inside the brain, and it is therefore impossible to differentiate radiopharmaceutical decay sites with brain parenchyma from those within blood vessels of the brain. To overcome this limitation, brain models with incorporated virtual vasculature were developed in this study to allow explicit differentiation of radioactive decay sites between brain tissue and brain blood vessels. Improved brain dosimetry via radionuclide *S*-value calculations using the blood inside the created vasculature are presented.

## 2. Materials and methods

The vessel generation algorithm based on the CCO method described by Correa-Alfonso et al (2022) is applied to generate virtual binary trees of the arterial and venous cerebral vasculature of the adult male and adult female mesh-type reference computational phantoms (MRCPs). The first sections describe the main characteristics of brain vasculature, the development of brain sub-regions within the adult reference brain surfaces using a multi-region brain model based on MRI data of a healthy human volunteer, and the creation of main cerebral arteries and veins that reach each sub-region using a well-detailed anatomical atlas (Nowinski et al 2015). In the following sections, the generation of vascular trees and the conversion from mesh-surface to the tetrahedral mesh format are described. The last section describes the internal dosimetry calculations based on the MIRD schema by defining dual-region brain models with explicit differentiation of decay sites within brain blood from those within brain tissue.

### 2.1. Main features of brain anatomy and vasculature

**Figure 1.**
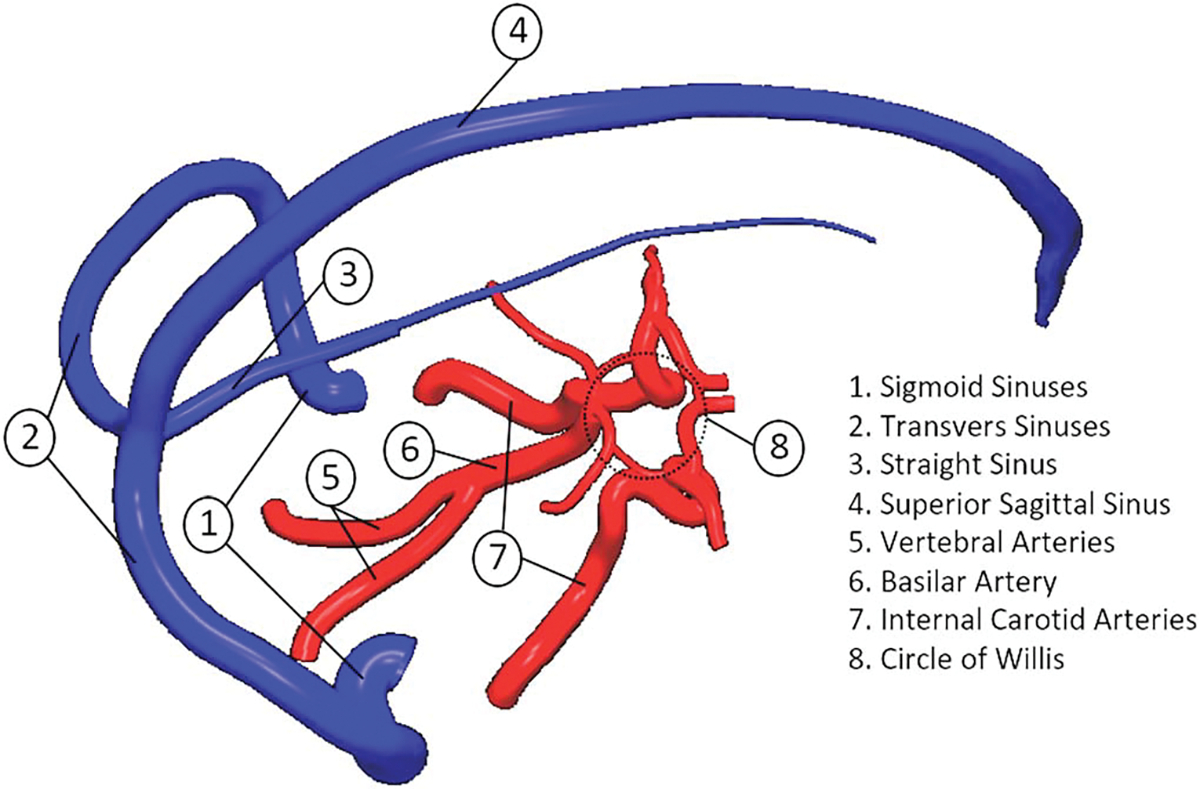
Main arteries (red) and veins (blue) of the brain vasculature. Illustration is based on the 3D anatomical atlas developed by Nowinski et al (2015).

Four large arteries supply the blood flow of the brain: one internal carotid artery (ICA) and one vertebral artery (VA) on each side of the brain. Typically, the ICAs on both sides are referred to as the anterior circulation, and the posterior circulation is defined by the vertebral-basilar arterial system. At the central base of the cranium, the anterior and posterior circulations connect via an anastomotic polygon system called the ‘Circle of Willis’ (CoW). The CoW is composed of the anterior cerebral arteries (ACA), the posterior cerebral arteries (PCA), the anterior communicating branch (AcA) (which connects both ACAs), and the posterior communicating arteries (PcA) (which link the ICA and PCA within each brain hemisphere) (Konan et al 2022). Figure 1 shows the main arteries and veins that supply and drain blood in the human brain.

Several arteries arising from the CoW travel along the brain surface and give rise to pial arteries, which branch out into penetrating arteries and arterioles. The penetrating vessels are slightly divided from the brain tissue by an extension of the subarachnoid space called the Virchow–Robin space. The penetrating vessels dive down into the brain tissue, giving rise to intracerebral arterioles, which ultimately branch into capillaries where the exchange of oxygen, carbon dioxide, nutrients, and metabolites between the blood and the tissues takes place (Hall 2016).

Normal blood flow through the brain of the adult person averages 50 ml per 100 g of brain tissue per min (Cipolla 2009). For the whole brain, this amount is about 750 ml min^−1^. Thus, the brain constitutes only about 2% of the body weight but receives 12% of the resting cardiac output (ICRP 2002).

The venous drainage of the cerebral hemispheres is guaranteed by two systems: (1) the superficial system, which reaches the peripheral dural sinuses (e.g. the superior sagittal sinus, the lateral sinus, and the cavernous sinus), and (2) the deep system, via the Galen’s vein (Meder et al 1994). Both these systems mostly drain into internal jugular veins. The veins draining the brain do not follow the same course as the arteries that supply oxygenated blood to the brain (Uddin et al 2006).

### 2.2. Development of brain sub-regions

The brains within the ICRP adult male and female mesh-type reference phantoms are represented as a single closed mesh surface with no delineation of brain sub-structures. To define vascular volumes of perfusion, the single-mesh surface brains were partitioned into several vascular territories. To define the vascular territories, a previously developed multi-region brain model created in our laboratory based on segmented 3 T MRI images of a healthy adult male and a healthy adult female volunteer (Long 2014) were utilized as templates to partition the adult male brain (AMB) and adult female brain (AFB) into 13 sub-regions.

The original UF multi-region brain models were initially segmented from MRI images (Long 2014). After segmentation, the resulting models had a significant number of intersections between the brain sub-structures as it was originally used in a voxelized format. In moving to tetrahedralized phantoms as opposed to voxel or simple polygon mesh phantoms, all intersections needed to be removed. First, mesh Boolean difference was applied between each brain sub-region and any of the others adjacent sub-regions and the homogeneous single-region mesh brain to guarantee that all brain sub-region are completely inside the single region brain. After this step, each resulting mesh brain sub-region was checked for the presence of any problems such as self-intersecting faces, non-manifold edges and duplicated faces. For each mesh sub-region, the ‘*ExtractNonManifoldMeshEdges*’ and ‘*ExtractDuplicateMeshFaces*’ commands in Rhinoceros v6^4^ were executed to find any non-manifold edges and duplicated faces, respectively caused by the Boolean difference operation previously applied. In those cases in which non-manifold edges and/or duplicated faces were detected, we manually repaired. Once all mesh sub-region are defined as closed mesh, with only 2-manifold edges and free of duplicated faces, they were imported individually inside the homogenous mesh reference brain.

**Figure 2.**
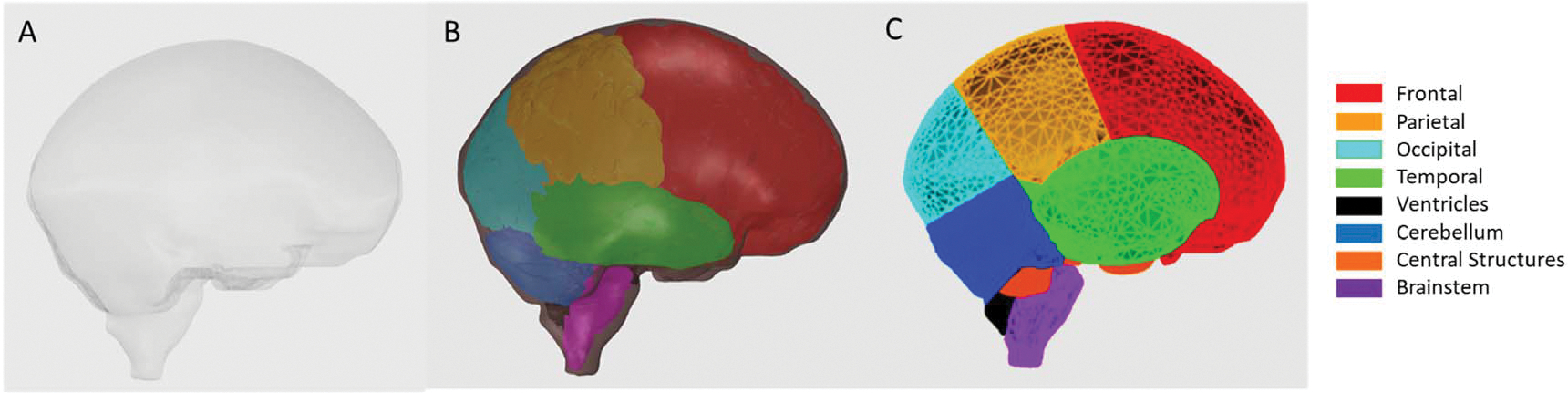
(**A**) Single mesh surface of AF-MRCP brain. (**B**) Sub-regions of the Multi-Region Brain model built-in the AFB mesh. (**C**) AFB is partitioned into 13 sub-regions based on the multi-region brain model.

As the focus of this study was not to provide highly detailed brain-sub-regions but to develop models of brain vasculature, the multi-region brain model was subsequently simplified. Once the models were fitted within the AMB and AFB, the boundaries between sub-structures could then serve as guides to subdivide the AFB and AMB into 13 brain sub-regions based on the 43 structures present in the multi-region brain model. To avoid introducing complex meshes as sub-regions, the boundaries between each subregion were defined using cutting planar meshes with minimal thickness passing through the boundaries of each sub-region. For those sub-regions where the boundaries could not be defined by planar structures, the meshes from the multi-region models were utilized after applying smoothing tools based on Laplacian methods (Field 1988) in Rhinoceros v6 and Space Claim,^5^ and Boolean operations were performed to define the sub-regions. The 13 vascular territories created are named as follows: left and right frontal lobes, brainstem, left and right parietal lobes, left and right temporal lobes, left and right occipital lobes, left and right portions of the cerebellum, and left and right central structures (which group several structures as the caudate nucleus, internal capsule, globus pallidus, putamen, thalamus and corpus callosum). Figure 2 shows the different stages of the development of the brain sub-regions in the AFB.

Percentages of total brain volume (PTBV) data from MRI studies of brain sub-regions volumes of human healthy adult male and female cohorts (Hammers et al 2003) were used as reference values to adjust each brain sub-region volume. Rigid deformation was performed in the Rhinoceros 3D scripting tool Grasshopper^™^ as applied to the AMB and AFB sub-regions in order to match the PTBV reference values. Table 1 shows the PTBV of the segmented AMB and AFB and the median PTBV values reported by the reference study.

### 2.3. Main vessels construction

In this study, main vessels refer to vessels that enter the brain from the neck and base of the brain to include the internal carotid arteries (left and right ICA) and transverse sinus (TS). In addition, there are blood vessels that feed and drain each brain sub-region. However, as dictated by the vessel generation algorithm, there can be only one artery and one vein that feed and drain blood, respectively, in each brain sub-region. To execute the algorithm to create vascular trees a brain sub-region previously created is needed to define the closed volume of vascularization. Another input required to run the algorithm is the starting point within each brain sub-region from which the algorithm will start creating the tree. To create the starting points, main vessels were manually constructed, starting from the ICAs and TS and going to their daughter’s vessels until they reach each brain sub-region. Once the main vessel vasculature is completed, each ending point of the branch of the main vessels in each brain sub-region was designated as starting point. With these starting points and the brain sub-regions already created manually, the algorithm can be executed successfully.

To construct the main vessels, geometrical parameters such as radii of all major vessels feeding to each of the brain sub-regions were recorded based on a well-established 3D anatomical atlas (Nowinski et al 2015). Radii values were extracted by taking an estimate of the average radius along the length of the vessel. These values were tabulated and used for both the AMB and AFB. To accommodate the higher blood volume of the AMB compared to AFB, radii values were scaled upward in proportion to the blood volume increment for the AMB. For veins, the first portion of the left and right transverse sinus, straight sinus, and superior sagittal sinus were scaled upward by a factor of 1.02 in the AMB.

A total brain blood flow rate of 50 ml min^−1^ per 100 g of brain tissue was considered as suggested by Cipolla (2009). Assuming the AMB and AFB have 1450 g and 1300 g of brain tissue (excluding blood), respectively, as indicated in ICRP Publication 89 (ICRP 2002), the cerebral blood flow would be 725 ml min^−1^ (for AMB) and 650 ml min^−1^ (for AFB). Using these values, the blood flow rates for the transverse sinuses were calculated to match the total outflow of the venous system (two transverse sinuses) to the total inflow of the arterial system. The total cerebral blood flow rate values utilized in this study for the AMB and AFB are in agreement with the values reported in healthy young adult individuals (Zarrinkoob et al 2015) as well as the venous blood flow rate value reported by Muller et al (1990).

Pressures of main arteries and veins entering the brain were extracted from reported studies in healthy adult cohorts (Bateman and Siddique 2014, Blanco et al 2017). Poiseuille’s law and conservation of blood flow rate were applied to find the blood flow rates and the pressure gradients down the root of the main entry vessels.

**Figure 3.**
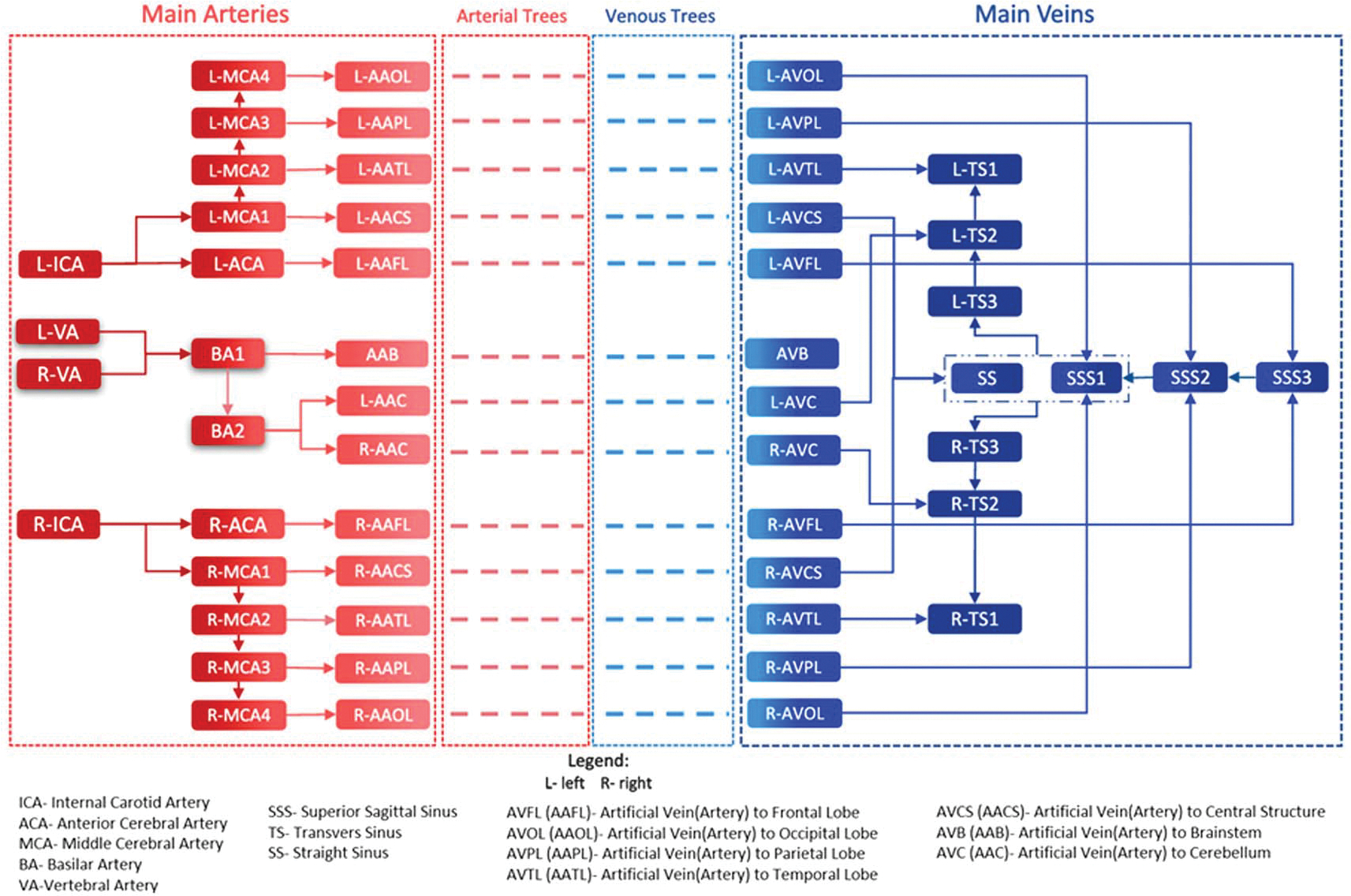
A diagram showing all of the main vasculature modeled prior to algorithmically generating vasculature within each brain sub-region.

With all the radii, pressure, and blood flow rates of main entry vessels obtained from the literature review (ICR 2002, Cipolla 2009, Bateman and Siddique 2014, Blanco et al 2017), the process of the calculation of a singular blood flow rate and radius for an ‘artificial’ vessel to each region could continue. With all the radii, pressure gradients and blood flow rates of the main entry vessels and its main daughter vessels, we were able to calculate the pressure, blood flow rate and radius of each branch of the main vessels (called ‘artificial’ vessels) from which each vascular tree will be developed. As our algorithm for vessel generation requires a unique entry point (which means a unique main vessel branch per brain sub-region) from which the vascular tree is created in each sub-region, but real brain vasculature in some sub-regions (i.e. frontal lobes) has more than one vessel that supplies/drain blood, we needed to account for all blood flow rates from all vessels that supply/drain a specific sub-region. Considering this fact, in each brain sub-region, we created one artificial artery/vein -as required by our algorithm- that carry the same amount of blood to/from a sub-region as several arteries/veins in the real brain sub-region vasculature. A diagram of the simplified main vessel network created in our brain models is shown in figure 3.

In the last step of the main vessel construction, Boolean union was performed to all main arteries and all main veins. Later, both structures were converted into mesh-surface types. Based on the 3D anatomical atlas representation of the head and neck presented by Nowinski et al (2015), the main veins and arteries that drain/feed the blood from each of the sub-regions were constructed.

### 2.4. Vascular tree generations in the adult male brain and the adult female brain

The algorithm was executed in Grasshopper, a graphic algorithm editor embedded inside Rhinoceros v6^6^. Several parameters and mesh objects are needed as inputs to run the algorithm. Meshes of the modeled main vessels, the centerlines of these main vessels, as well as the meshes of the brain sub-regions and the whole mesh reference adult brain (ICRP 2002), are imported into Grasshopper. Values of blood flow rates and pressures at the endpoint of each main vessel entering its corresponding brain sub-region were also incorporated (ICRP 2002, Cipolla 2009, Bateman and Siddique 2014, Blanco et al 2017). Lastly, the algorithm needs the terminal pressure at the very end of the tree it will generate. For terminal arterial pressure, an average value reported by Blanco et al (2017) for arterioles (approximately 0.1 mm radius) was utilized. For terminal venous pressure, 15 mmHg was used according to the value for the venular trunk pressure as reported by Lorthois et al (2011).

Once all the parameters and regions needed for the vessel generation are incorporated in Grasshopper, the algorithm begins by uniformly populating each of the brain sub-regions with a set number of bifurcation points. Next, the algorithm ensures that each of these points is both inside the specified mesh sub-region and not inside any of the void structures (main vessel meshes). Therefore, an amount larger than the desired number of generated random points per subregion must be chosen, since many are eliminated by not being within the correct region. The desired amount of generated random points per subregion is found by first determining an overall density of points resembling the detail of the vasculature. The selection of the number of terminal points will impact the overall amount of vessels constructed and the average minimum radius of the vasculature. In our previous work on liver (Correa-Alfonso et al 2022), selecting 1000 points was sufficient to create a vasculature between 0.1 and 0.2 mm radius. A high number of terminal points may produce a very detailed vasculature but could be a potential issue when importing into Monte Carlo radiation transport codes due to the high number of tetrahedrons. Under the assumption that regions with more volume and more blood flow rate are more vascularized, the number of terminal points per subregion was selected according to the PTBV value for each subregion. This assumption ensures an equal terminal blood flow rate at each terminal vessel for all vascular trees created in each adult reference brain.

Once the points are generated, all terminal points are ordered based on their proximity to the entry point of the tree and the centerlines are created, starting at the entry point and extending iteratively throughout the list of ordered points. At each iteration, and assuming the new candidate vessel is accepted, the vessel is added and vascular radii values throughout the tree are updated as each vessel is created iteratively in order to preserve the conservation of blood flow, Murray’s Law, and Poiseuille’s Law. The algorithm stops when all terminal points are connected to the tree.

**Figure 4.**
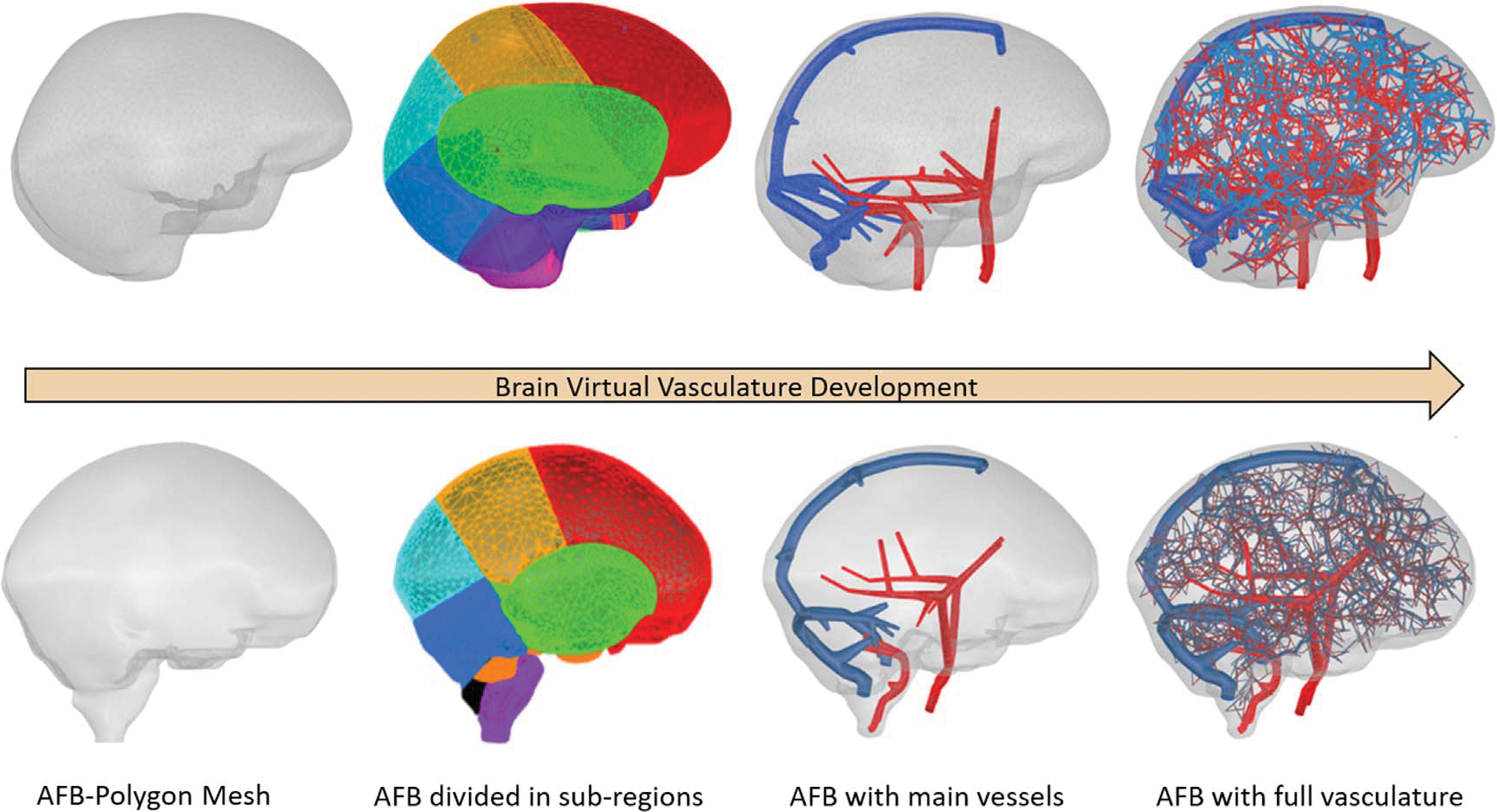
Different stages of the brain vascular model developed for the AMB and AFB.

Vascular networks of 4000 vessels distributed in 26 trees (13 arterial and 13 venous) were constructed inside the AMB and AFB. Figure 4 shows the different stages of the development of the brain vascular models inside the AMB and AFB starting from the outer surface brain meshes, then later defining the sub-regions of vascularization, incorporating the main vessels manually created and finally incorporating the vascular tree models generated for both adult reference brains (AFB and AMB).

**Figure 5.**
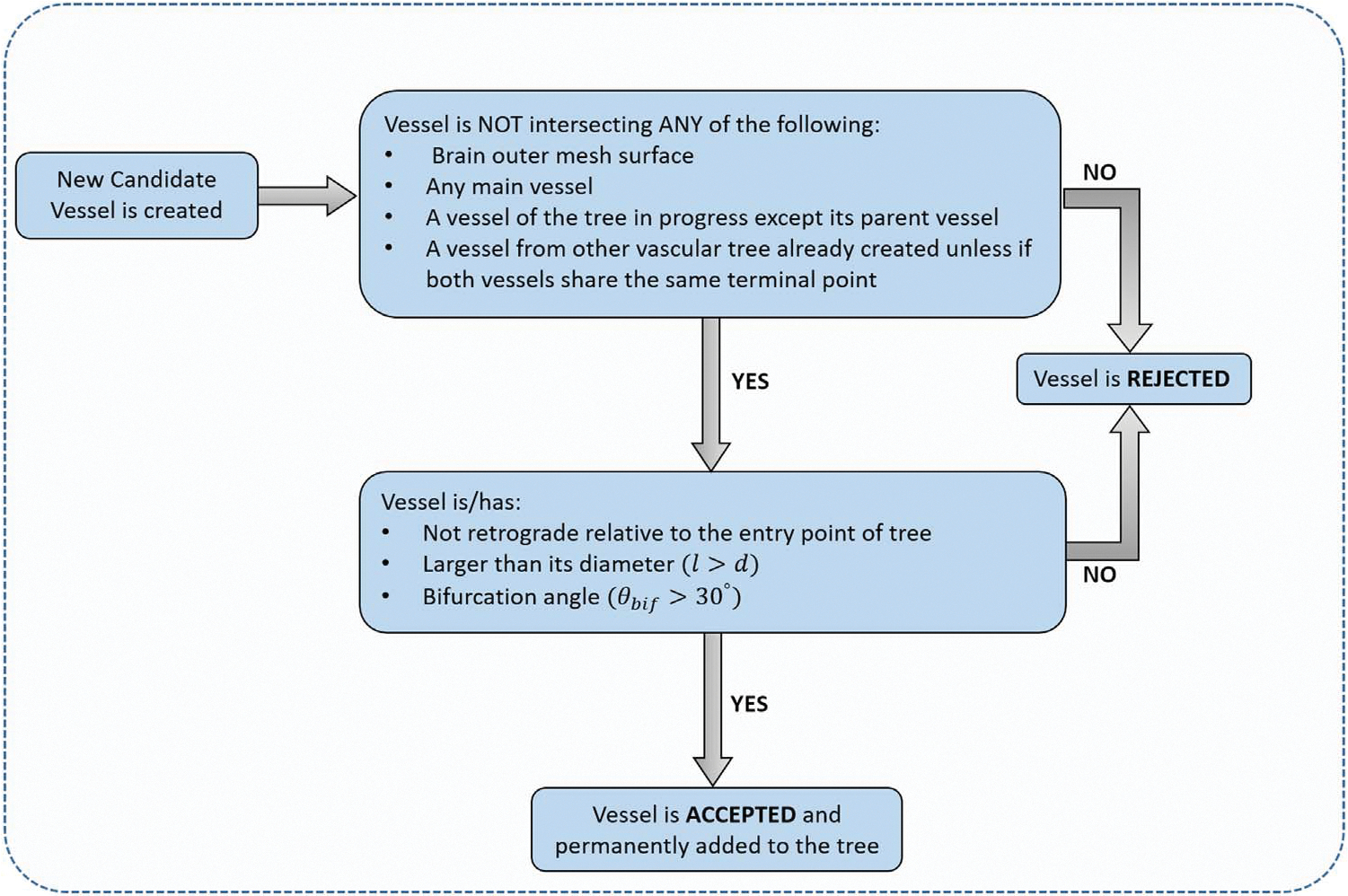
Sequential order followed within the algorithm to accept or reject a new generated vessel based on the appearance of intersections and the spatial location of the vessel relative to tree in progress.

To avoid the creation of vascular trees with intersecting vessels, the same approach as detailed in Correa-Alfonso et al (2022) was followed. Several ‘high-importance’ restrictions are considered in the optimization process within the algorithm to ensure that any new vessel generated will not intercept any structure already created in our model. In addition, the new vessel must satisfy several conditions to avoid the formation of retrograde pathways in the vascular trees and self-intersections between parent and daughter vessels which is achievable by defining a threshold bifurcation angle of 30 degrees, consistent with the minimum bifurcation angle reported by several studies based on morphometric analysis of the vasculature network of post-mortem adults (Hutchins et al 1976, Canham and Finlay, 2004). Figure 5 summarizes the process to accept or reject each vessel generated in the algorithm.

To create the whole brain vasculature, the algorithm is executed at each sub-region to create venous trees first. Each generated vessel of the venous trees are incorporated into the algorithm as voids regions. Thus, when generating arterial trees, any intersection with a new arterial vessel with any void region will be avoided. Only the intersections of a new arterial vessel with a terminal venous vessel that share the same terminal point are accepted. Otherwise, the new arterial vessel will not be added permanently to the arterial tree. The venous trees are selected to be generated before the arterial trees as veins usually have larger radii than arteries. This decision on creating the larger volumetric trees first resulted in less computational time as the number of iterations in which no vessels were accepted is reduced.

Although the algorithm optimizes vessel tree growth and factors in the aforementioned constraints, sometimes intersections between generated branches, intersections with generated branches and the outer surface of the brain, or intersections between generated branches and main vessels can occur when the remaining points limit the ability of the algorithm to both optimize the tree and preserve Poiseuille’s Law. In these cases, intersections are manually corrected as a post-processing step in vessel generation. The manual correction of intersecting vessels was done first by performing small translation of terminal point of one of the vessel that cause the intersection. If translating any of the terminal points of the intersecting vessels does not fix the intersection, then we performed rotation of any of the intersecting vessel around its corresponding bifurcation point. In the rare case in which neither translation of terminal point nor rotation of vessel around its bifurcation point fixed the intersections, both transformations (rotation and translation) were done sequentially in one or all the vessels intersecting. In all cases, intersections were eliminated with minimal changes to the vascular tree.

### 2.5. Creation of tetrahedral brain models

Each vessel of the AMB and AFB vasculature was represented as independent rigid mesh pipes. To create smooth connections between all individual vessel pipes, the individual pipes were created with rounded, as opposed to flat, ends. The next step was to create a single mesh that joins all vasculature trees which involved the use of Boolean union tools. Space Claim was utilized and each vascular network represented as individual mesh pipes was grouped and exported from Rhinoceros as ‘*.obj’ files and imported into Space Claim. Automatic mesh repair and mesh face reduction tools were cautiously performed to the imported ‘*.obj’ files in Space Claim software to avoid the creation of a bad^7^ mesh and over-detailed mesh of the AMB and AFB vascular models. The mesh faces reduction tool was applied iteratively. At each iteration, the mesh faces were reduced by 10% and the mesh volume and the presence of errors in the resulting mesh were checked. Finally, the mesh faces were reduced to the minimum amount of faces needed to reproduce the whole vasculature without any volume changes or introducing errors in the mesh. The final mesh models of the AMB and AFB vasculature were exported as ‘*.obj’ files and imported back into Rhinoceros to be incorporated inside their corresponding brain mesh surfaces.

To apply the brain models enhanced with the vascular network for radiation dosimetry studies, it was necessary to convert the models to either tetrahedral models or voxel models as these two formats are both compatible with the PHITS radiation transport code. As mesh faces can be as small as 0.01 mm, conversion to voxel format will not preserve the overall shape of the vasculature (especially in the vessel junctions). On the other hand, tetrahedral mesh (TM) models offer many advantages. TMs not only facilitate fast computational speeds and inhomogeneous density representation as well as the voxelized models, but they are also deformable, flexible, and preserve surface smoothness (which is not possible to achieve when using voxel models). TMs preserve the entire structure of the polygon mesh surface models and also improve the computational speeds (Yeom et al 2014) compared to voxelized phantoms. With all the advantages of TMs over voxelized models, it was decided in this study to convert the AMB and AFB created from surface mesh to tetrahedral mesh format. This conversion was performed using the POLY2TET software (Han et al 2020).

**Figure 6.**
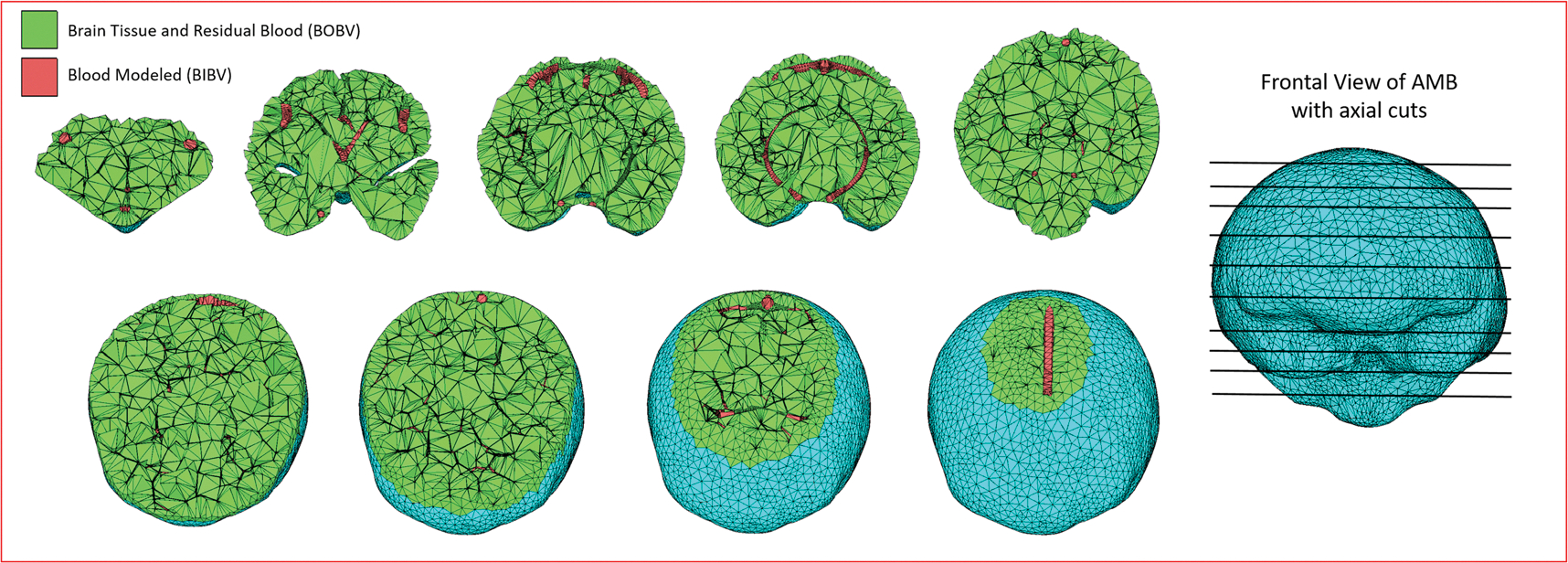
Several axial cuts of the tetrahedral mesh-type model of the AMB with incorporated vasculature. Red tetrahedrons represent the vascular model generated (BIBV) and green tetrahedrons are a homogenous mixture of residual blood and brain tissue (BOBV).

The AMB and AFB with the vascular networks were exported from Rhinoceros in OBJ format and different organ tag numbers were assigned to the vascular region and the brain mesh outer surface. POLY2TET was executed and tetrahedral mesh models were created. Visualization of the tetrahedral AMB model (figure 6) at different axial cuts showing the two regions (inside and outside the vasculature) was done using TETVIEW^8^,a graphic program for visualizing tetrahedral meshes.

### 2.6. Dual region brain models for radiopharmaceutical therapy applications

The MIRD schema (Bolch et al 2009) states that organ absorbed dose in radiopharmaceutical dosimetry is calculated as the product of the time-integrated activity $\tilde{A}\left(r_{S}\right)$ (total number of decays within the source region) of the radionuclide and the mean absorbed dose $S\left(r_{T}\leftarrow r_{S}\right)$ to the target region $r_{T}$ per nuclear decay in the source region $r_{S}$. The *S*-value is then computed as

(1) $$S\left(r_{T}\leftarrow r_{S}\right)=\sum_{i}\;E_{i}Y_{i}\Phi \left(r_{T}\leftarrow r_{S},E_{i}\right),$$

where $E_{i}$ and $Y_{i}$ are the energy and yields of the i-th nuclear transformation of the radionuclide, respectively. The term $\Phi \left(r_{T}\leftarrow r_{S},E_{i}\right)$ is the specific absorbed fraction (SAF) for a radionuclide particle of energy $E_{i}$ for a given source-target combination. The SAF is also computed as the quotient of the absorbed fraction (AF) and the target mass

(2) $$SAF=\Phi \left(r_{T}\leftarrow r_{S},E_{i}\right)=\frac{AF\left(r_{T}\;\leftarrow \;r_{S},\;E_{i}\right)}{m\left(r_{T}\right)}.$$

The existing approach to calculate SAF and *S*-value considers that organs within the anatomical patient models are modeled as single-region volumes where intra-organ blood and parenchymal tissue are homogeneously combined. Using that approach, a single-region brain model (B) considers the brain parenchyma (BP) and the blood within the brain (BB) as a homogenous region of mass $m_{B}$ uniformly distributed within the brain volume. Using this single-region brain model, the SAF values are calculated as

(3) $$SAF\left(B\leftarrow B\right)=\frac{AF\left(B\;\leftarrow \;B\right)}{m_{B}}.$$

For the ideal scenario in which BP is both target and source, the SAF value is defined as SAF(BP←BP). Similarly, for the case in which BB is the source and BP is the target, the SAF is defined as SAF(BP←BB). Under the single-region brain model, the only approximation to both SAF values mentioned above is achievable by using equation (3). Given the lack of spatial differentiation of the location of blood within the brain, the main assumption used in the SAF calculations for the single-region model presented in our study is that the only source ← target combination that can be employed to compute SAF(BP←BP) and SAF(BP←BB) is by using B←B as a source-target combination as this homogeneous mixture of brain tissue and brain blood is the only tissue defined in the single-region brain model. This assumption is valid as there are negligible differences between the mass energy-absorption coefficients of blood (BB) and brain parenchyma (BP), and the mixture of blood and brain parenchyma (B) obtained from the National Institute of Standards and Technology^8^ (NIST) database. Also from NIST, the CSDA range of electrons within the energies studied are very similar for B compared to both BB and BP with negligible maximum relative percent differences of 0.87% and 0.1%, respectively. Thus, SAF(B←B) was used for the single region brain models as approximation of the SAF(BP←BP) and SAF(BP←BB) as defined in equation (4). This approach is the current basis for all internal dosimetry calculations using existing computational phantoms which do not include intra-organ blood vessel models (ICRP 2016). With the AMB and AFB vascular models presented in this study, more refined approximations for SAF(BP←BP) and SAF(BP←BB) are possible within the dual-region brain model

(4) SAF(BP←BB)≈SAF(BP←BP)≈SAF(B←B).

The dual-region brain model is defined by two regions: blood inside blood vessels modeled (BIBV) and residual blood not modeled but homogenized with the brain outside of blood vessels (BOBV). Under the dual-region model, it is possible to obtain refined SAF values for the following target and source combinations: (BP←BP) and (BP←BB). For the dual-region brain models, and as analogue to equation (4), the values of SAF(BP←BP) and SAF(BP←BB) will be approximated as

(5) $$\begin{array}{l} SAF(BP\leftarrow BP)\approx SAF(BOBV\leftarrow BOBV)\\ \;=\frac{AF(BOBV\;\leftarrow \;BOBV)}{m_{BOBV}} \end{array}$$

(6) $$\begin{array}{l} SAF(BP\leftarrow BB)\approx SAF(BOBV\leftarrow BB)\\ =\frac{f_{BV}\;\cdot AF(BOBV\;\leftarrow \;BIBV)\;+\;\left(1\;-\;f_{BV}\right)\;\cdot AF(BOBV\;\leftarrow \;BOBV)}{m_{BOBV}}, \end{array}$$

where $m_{BOBV}$ is the mass of the BOBV region and $f_{BV}$ is the fraction of total brain blood that is modeled explicitly in both manually created and algorithmically generated blood vessels. In equations (5) and (6), the approximation sign reflects the fact that although the dual-region brain model incorporate a considerable amount of the reference blood within the adult (male/female) reference brain via explicit vascular models, there still remains residual blood stored in very small vessels and capillaries that are not modeled and thus must be considered in the BOBV region.

Although the AMB and AFB vascular models account for 47% and 56% of the total blood content in the adult reference brains, respectively, accounting for these fractions of the total brain blood model would have an impact on internal dosimetry. It is expected to have some difference from the single-region approximations specifically for short-range particles that can deposit their energy partially or completely inside the vascular model (blood self-dose).

PHITS radiation transport simulations using the University of Florida HiPerGator computing cluster were performed to compute AF values in the dual-region brain model. Monoenergetic alpha particles, electrons, and photons were defined as sources using the single-region brain model and independently in both BIBV and BOBV regions in the dual-region brain model. Particle sources were randomly sampled within the homogenized region in the single-region brain model. In the dual-region brain model, two independent simulations were performed in which the particle sources were uniformly distributed in either the BIBV or BOBV regions, respectively. For alpha particles, a total of 24 energies ranging from 0.5 to 12 MeV were sampled on a linear scale in increments of 0.5 MeV. For electrons and photons, a total of 26 energies were used in a logarithmic energy grid from 10 keV to 10 MeV. In all simulations, enough particle histories were generated to guarantee relative errors in energy deposition tallies below 1% for both single and dual-regions. Details of the Monte Carlo simulations performed are summarized in table 2.

The elemental compositions and mass densities were calculated for all regions defined in both the single-region and dual-region models. Table 3 provides the percentage of mass for each element that defines the brain parenchyma (BP), brain blood (BB and also BIBV), brain parenchyma mixture with blood not modeled outside the modeled blood vessels (BOBV), and the fully homogenized brain of the single-region model (B).

Emission energies and yields were taken from the MIRD Monograph on Radionuclide Data and Decay Schemes as given in the MIRD-07.RAD and MIRD-07.BET datafiles (Eckerman and Endo 2008). For beta particles, the complete energy spectra were considered instead of considering only their mean energies. For positrons, computed SAFs for monoenergetic positrons (collisional component only) were computed and compared to electron SAFs. The percent differences between both SAFs were less than ~0.2 %, thus electron SAFs were employed in the calculation of S-values for all positron emitters. *S*-values were calculated using a Python script that interpolates through particle energies using piecewise cubic Hermite interpolation polynomials (PCHIP). *S*-values for photons, beta particles, electrons, alpha particles, and alpha recoil particles were computed. For the alpha recoil particles, a method previously adopted by Publication 133 (ICRP 2016) in which the SAF values were interpolated at 2 MeV alpha particles was used. For alpha-emitter decay chains, *S*-values for the parent radionuclide and all individual progeny were calculated and reported individually.

## 3. Results

### 3.1. Morphometric analysis of adult male and female brain vasculature

The Strahler order is commonly utilized to analyze the morphometry of virtual vascular trees. To perform a morphometric analysis and qualitatively compare the vascular models created against other virtual models, the distribution of the number of vessels created by Strahler order in both arterial and veins networks was obtained.

**Figure 7.**
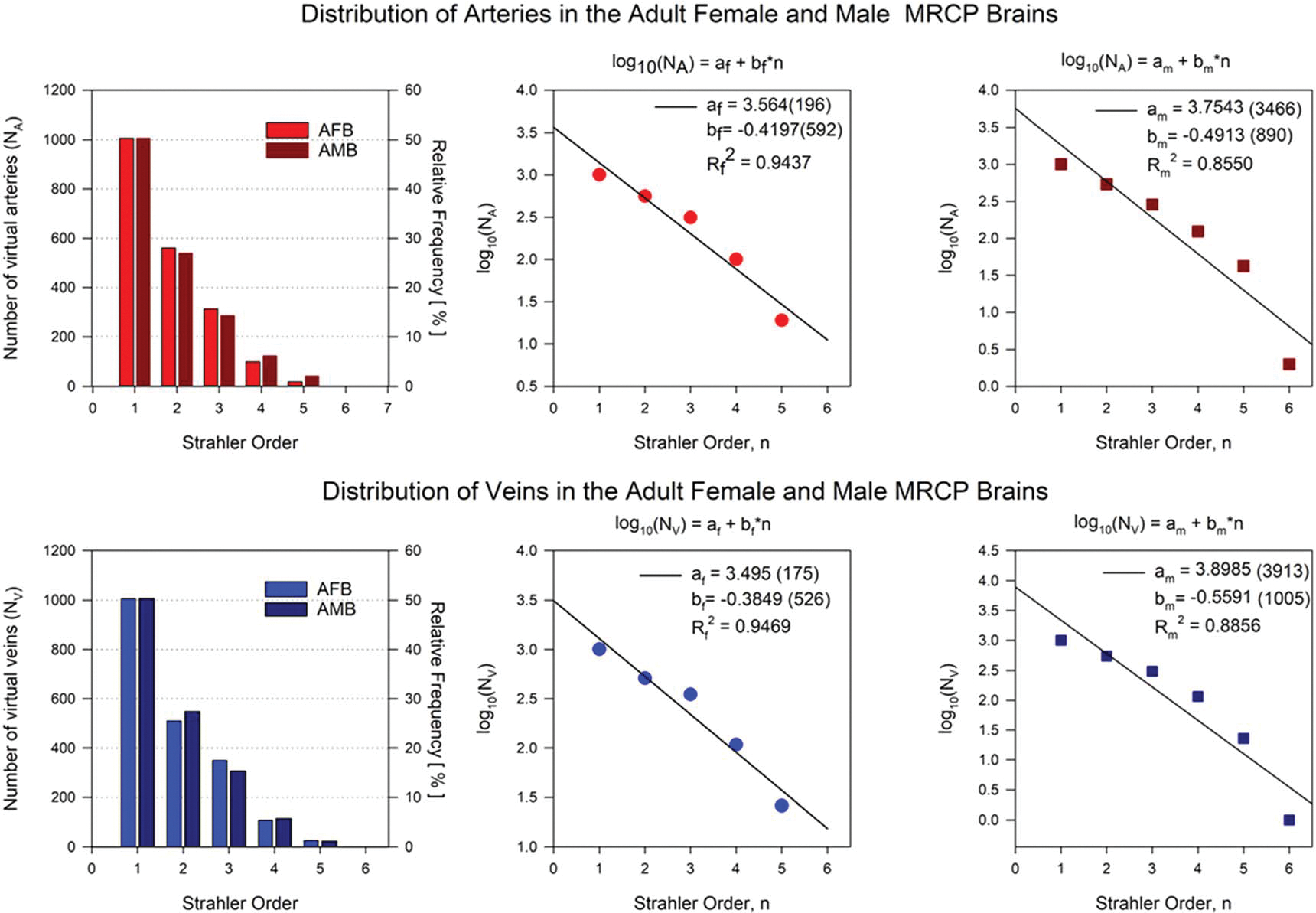
Distribution of virtual arteries and veins in the vascular trees per Strahler Order in AMB and AFB.

All terminal vessels were assigned a Strahler order of one. Only if a bifurcation vessel has two daughter vessels with the same Strahler order, the Strahler order of the bifurcation vessel is increased by one compared to the Strahler order of the daughters. In cases in which the daughter’s vessels have different Strahler orders, the highest of the Strahler order of the daughters will be assigned to the bifurcation vessel. Figure 7 shows the distribution of arteries and veins of AMB and AFB vascular models as a function of the Strahler order. Exponential decay fits of the number of vessels *N* per Strahler order—in terms of log(*N* ) are shown in figure 7.

**Figure 8.**
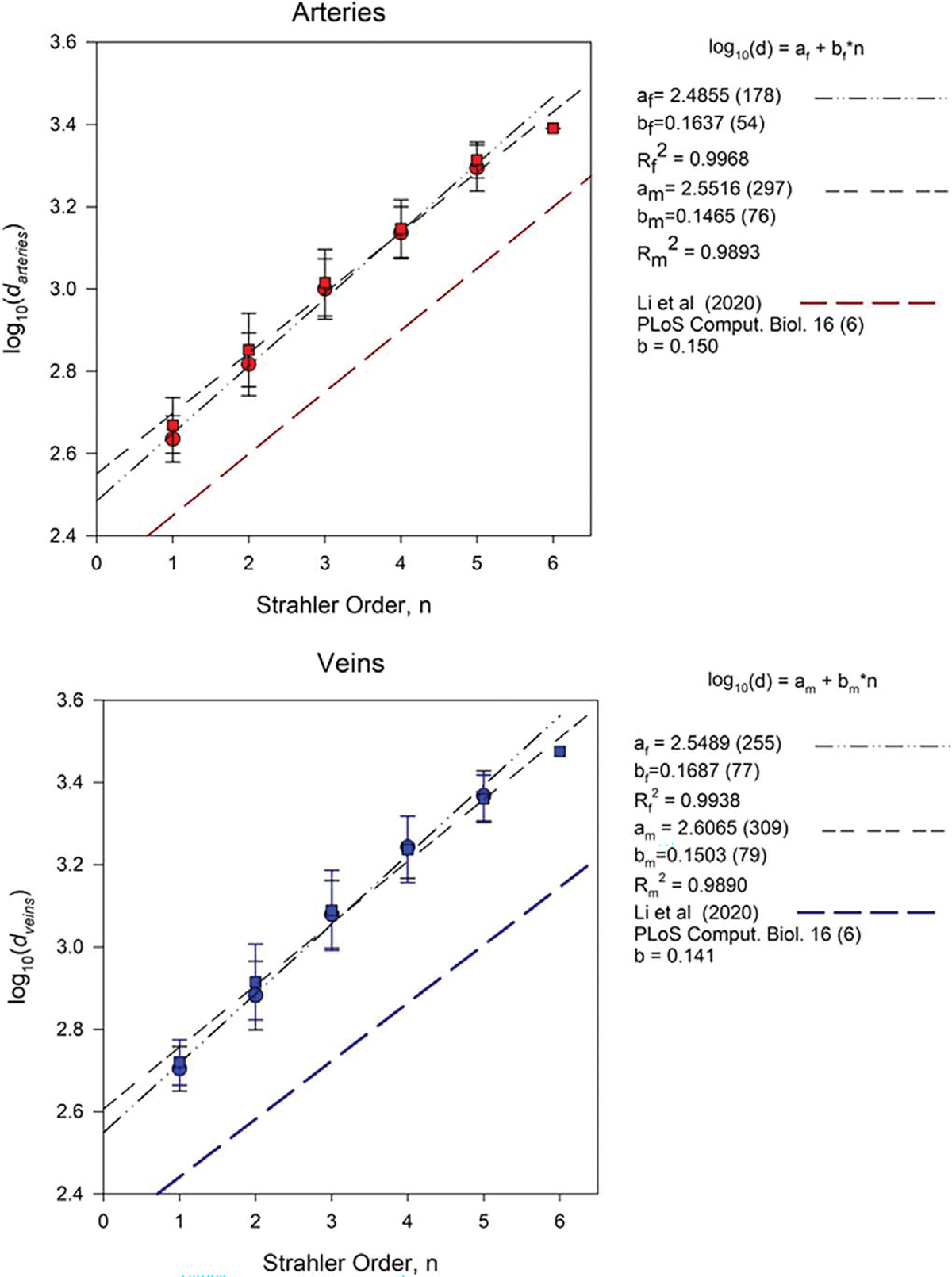
Morphological features of the mean diameters for arteries(top) and veins(bottom) as a function of Strahler Order for the AMB (squares) and the AFB (circles). Red symbols are assigned to arteries and blue to veins.

Another parameter in this morphometric analysis is the mean vessel diameter ‘*d*’ as a function of the Strahler order. Figure 8 displays the mean diameter—in terms of log (*d*)- obtained for all arteries and veins with equal Straher order. Linear fitting of the log (*d*) for arteries and veins of the AMB and AFB are also included in figure 8.

**Figure 9.**
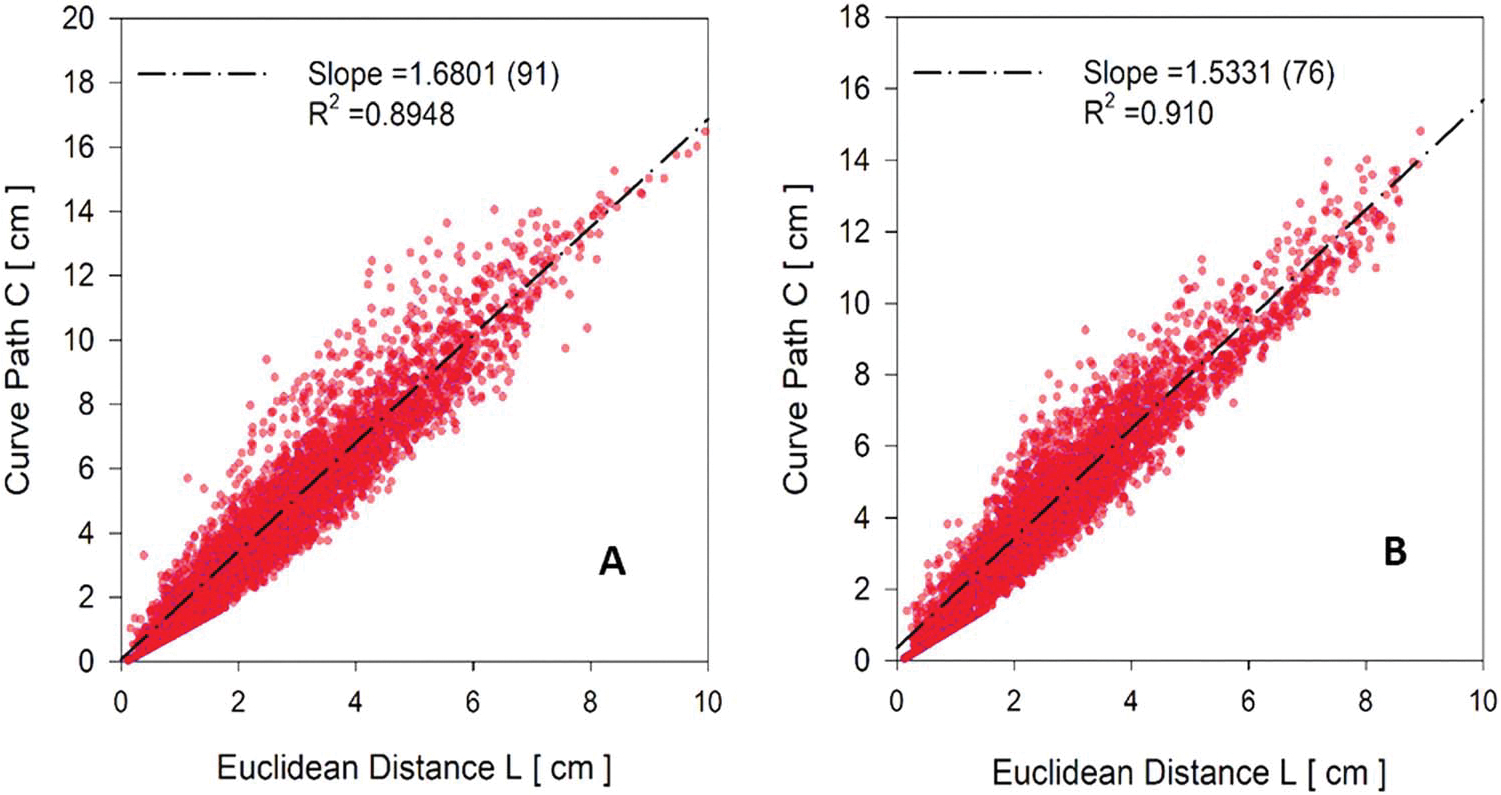
Distribution of curve path and Euclidean distance from each vessel to each main vessel branch in the whole vasculature of AMB (**A**) and the AFB (**B**).

The last parameter analyzed was the curve path and the Euclidean distances. The former is defined as the distance from the end of each vessel to the entry point of the tree by following the path within the vascular centerlines. The latter is defined as the straight line that connects each terminal vessel’s endpoint to the entry point of the tree. Curve paths of all generated vessels as a function of their Euclidean distances were plotted for the whole vasculature of the AMB and AFB as shown in figure 9.

### 3.2. Brain internal dosimetry

Using the single and dual-region brain models, Specific absorbed fractions (SAF) were calculated as shown in equations (3) and (5) and (6) respectively. In the dual-region brain model, blood decays were modeled from two sites: (1) sites within explicitly modeled blood vessels (BIBV) and (2) blood not modeled but mixed with brain parenchyma (BOBV).

**Figure 10.**
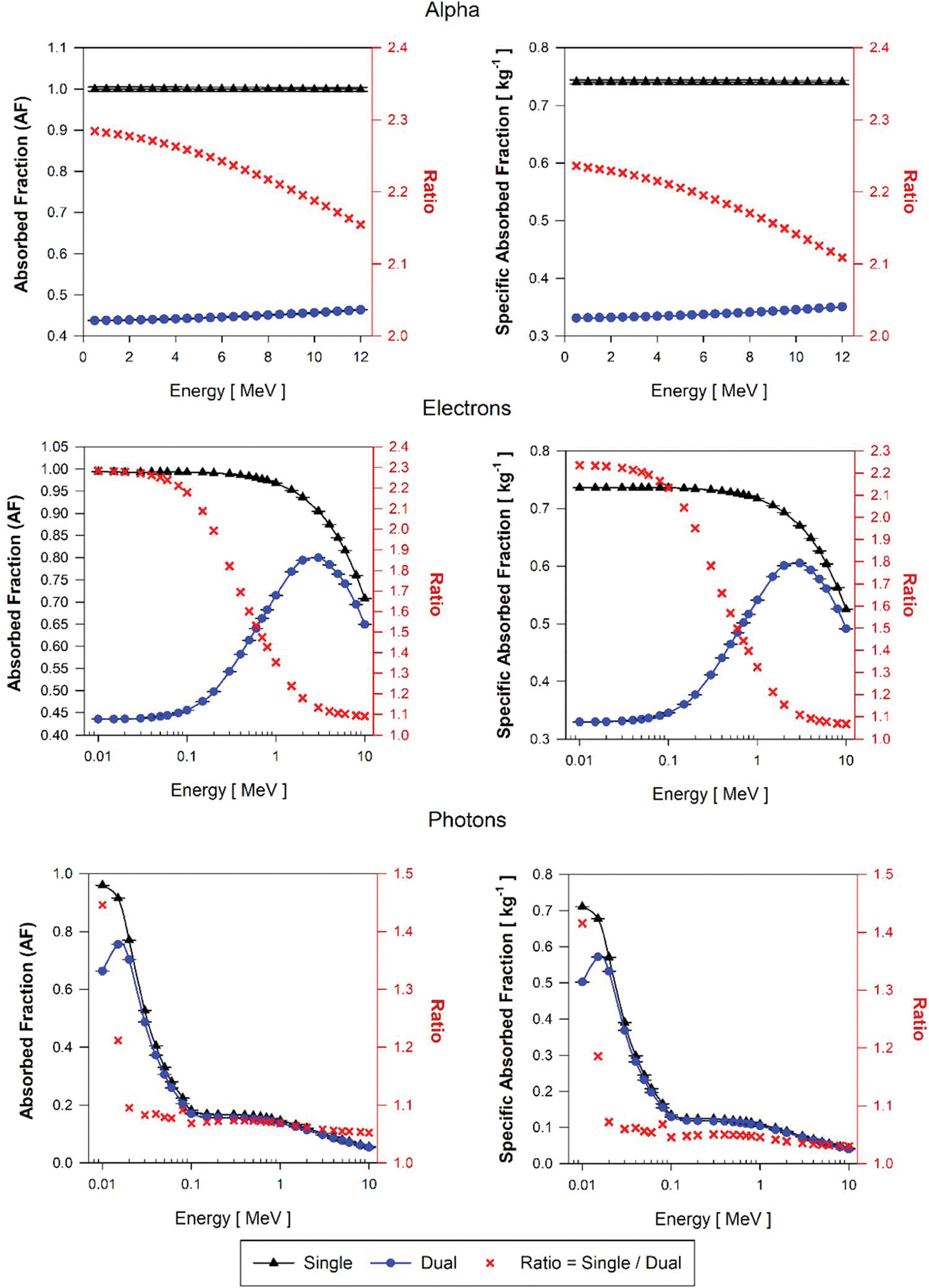
Approximations of AF(BP←BB) and SAF(BP←BB) for monoenergetic alpha particles(top), electrons(center), and photons(bottom) using the single-region brain model (black triangles) and the dual-region brain model (blue circles) in tetrahedral mesh-type format for the reference adult female brain (AFB).

**Figure 11.**
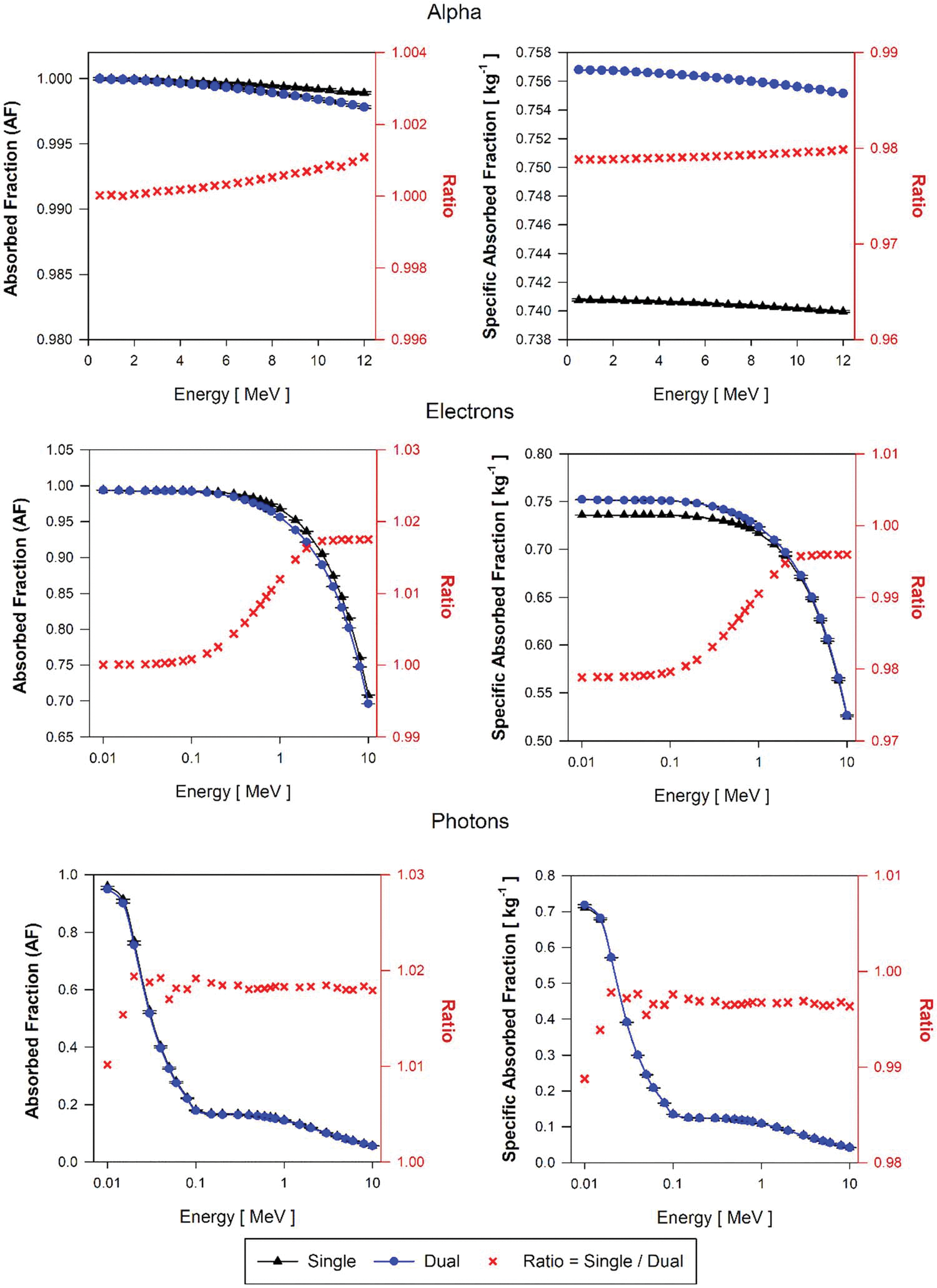
Approximations of AF(BP←BP) and SAF(BP←BP) for monoenergetic alpha particles(top), electrons(center), and photons(bottom) using the single-region brain model (black triangles) and the dual-region brain model (blue circles) in tetrahedral mesh-type format for the reference adult female brain (AFB).

**Figure 12.**
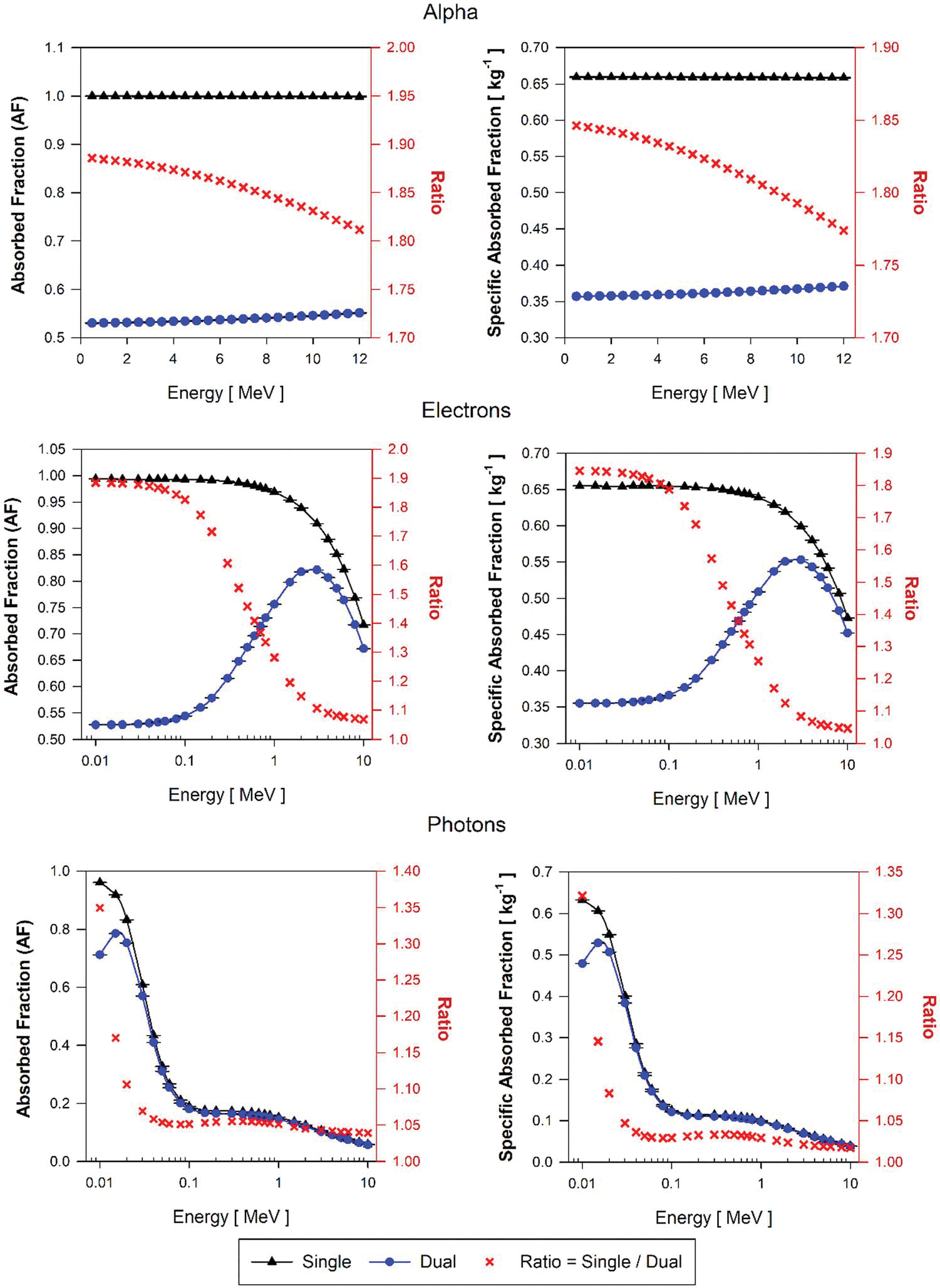
Approximations of AF(BP←BB) and SAF(BP←BB) for monoenergetic alpha particles(top), electrons(center), and photons(bottom) using the single-region brain model (black triangles) and the dual-region brain model (blue circles) in tetrahedral mesh-type format for the reference adult male brain (AMB).

**Figure 13.**
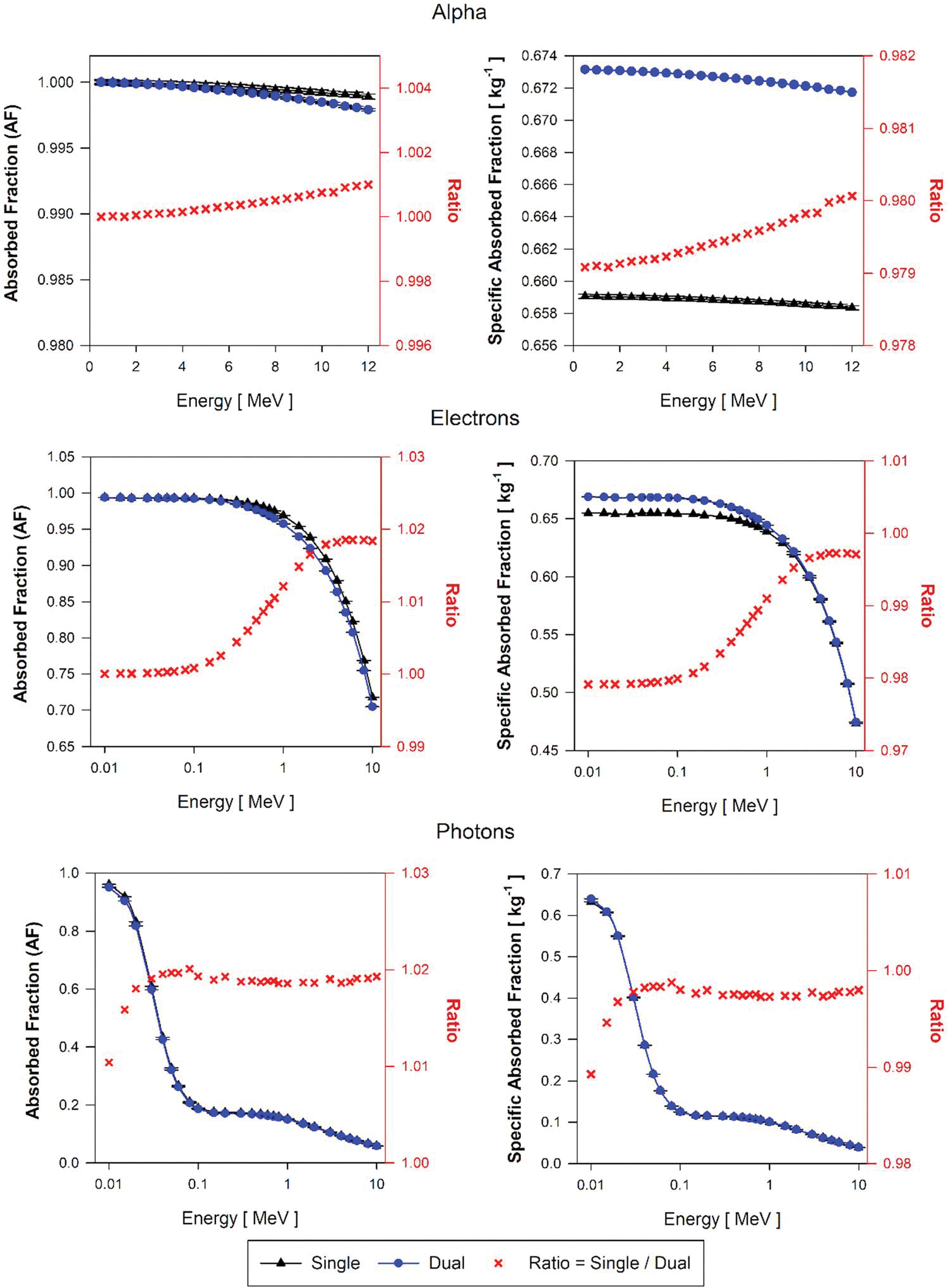
Approximations of AF(BP←BP) and SAF(BP←BP) for monoenergetic alpha particles(top), electrons(center), and photons(bottom) using the single-region brain model (black triangles) and the dual-region brain model (blue circles) in tetrahedral mesh-type format for the reference adult male brain (AMB).

Figure 10 displays the AF and SAF values for alpha particles, electrons, and photon sources emitted within brain blood (BB) in both single-region and dual-region tetrahedral mesh models of the AFB. The ratio of values (AF and SAF) from single and dual-region models is also included in figure 10. Figure 11 shows AF and SAF values for single and dual regions and the ratio of values from single and dual but considering the decay sites in the brain parenchyma (BP) for the AFB. Corresponding plots are also shown in figures 12 and 13 for the AMB, respectively.

## 4. Discussion

Figure 7 shows the number of vessels (N) created per Strahler order (n) for the arterial and venous trees in both AMB and AFB. The distributions of vessels for the AMB and AFB vascular trees follow very similar decreasing trends and exhibit exponential decay tendencies with the increase of the Strahler order. The number of arteries $N_{A}$ and veins $N_{V}$ shown in the histogram plots in figure 7 were expressed in terms of $log\left(N_{A}\right)$ and $log\left(N_{V}\right)$. These log frequencies were fitted to linear decreasing functions log(N)=a+b⋅n. The slopes b obtained for the AFB and AMB arterial trees were −0.420 and −0.491 respectively and is in good agreement with the slope (b=-0.461) reported by Ii et al (2020). The slope (b=-0.441) for the venous vasculature reported by Ii et al (2020) is very similar to those reported in figure 7 of our study for both AFB (b=-0.385) and AMB (b=-0.559) venous trees. In addition, our slopes are in good agreement with the slope (b=-0.361) reported by Lapi et al (2008) for the arterial network of rat. The coefficients of determination (*R*^2^) obtained in our fits were greater than 0.94 and 0.85 for arteries and veins, respectively.

Figure 8 shows the log frequency of the mean diameter of arteries and veins in the AMB and AFB. A linear increasing relationship of the mean diameter with the increase of the Strahler order is noticeable in all cases. The log frequencies were fit to linear functions log(d)=a+b·n and the slope b was compared to morphometric data reported (Ii et al 2020). The linear fits for arteries were very similar between AMB and AFB, and the same behavior was noticed for the linear fit in the veins of the AMB and AFB. In all cases, coefficients of determination were higher than 0.98. As shown in figure 8, slopes of 0.164 and 0.147 were obtained for the arterial trees of the AFB and AMB, respectively. For the venous trees, slopes of 0.1169 and 0.150 were obtained for the AFB and the AMB models. Our values are in good agreement with the slopes (b=0.150 for arteries and b=0.141 for veins) reported by Ii et al (2020) and the value of b=0.146 obtained by Lapi et al (2008) for rat pial arterial network.

Figure 9 plots each vessel curve path and Euclidean distance for both AMB and AFB. The study reported by Ii et al (2020) has shown that these quantities are linearly proportional. Thus, a linear regression line is also plotted for the AMB and AFB vasculature. Coefficients of determination higher than 0.89 were obtained for both regressions and similar slopes (b=1.53 for AFB and 1.68 for AMB) were obtained in comparison with the slope ( = 1.75) reported by Ii et al (2020).

Figure 10 shows AF(BP←BB) and SAF(BP←BB) for the AFB single and dual-region models. For alpha particles of all energies, the ratio of the AF value computed using the single-region brain model and the dual-region brain models is 2.3 at the lowest alpha energies and decreases to 2.15 at 10 MeV alpha energy. This ratio shows how the energy deposition in blood parenchyma is reduced by using explicitly modeled blood vessels as the dual-region brain models in which alpha particles can deposit a considerable amount of energy within the blood. Correspondingly, values of SAF(BP←BB) in the single-region model are between 2.21 and 2.1 higher than those SAF given by the dual-region brain model. The SAF values are obtained as the quotient of the AF and the target mass. The mass of the single-region AFB model ($m_{B}$) is 2.1% higher than the target mass ($m_{BOBV}$) in the dual-region AFB model. Thus, changes in target mass only partially account for these SAF percent differences.

For low-energy electrons (less than 100 keV) in the AFB, AF(BP←BB) calculated using the single-region model are on average 2.25 times higher than those obtained with the dual-region brain model. Above 100 keV and up to the maximum energy simulated (10 MeV), the ratio of AF(BP←BB) values in the single-region to values in the dual-region models decreases from 2.2 to 1.1. SAF(BP←BB) values using the single-region brain model are up to 2.23 times higher than those of the dual-region brain model for electrons below 100 keV. In the dual-region brain model, very low-energy electrons generated in the BIBV region deposit all the energy locally in the region and thus do not deposit energy in the BOBV region. This energy deposition differentiation between parenchyma and blood is impossible to discern in the single-region brain model as there is no differentiation between brain tissue and brain blood. With the increase of electron energies, the probability of these events–of full energy deposition in blood—decreases because the electron range increases in relation to the size of the blood vessel modeled (BIBV region). Furthermore, an increase in electron energy, comes along with the increase of bremsstrahlung radiation emissions, resulting in an additional decline in local blood self-dose.

For photons, the values of AF(BP←BB) obtained using the single-region brain model are up to 45% greater than the AF(BP←BB) values using the dual-region at the lowest photon energies simulated (about 10 keV). The ratio of AF(BP←BB) values in both models decreases drastically between 10 and 20 keV and continues decreasing but with a very small slope from 1.1 to 1.05 for photon energies between 30 keV and 10 MeV. The photon SAF(BP←BB) ratio is 1.4 at the lowest energy (10 keV) and decreases drastically between 10 and 20 keV to a ratio of 1.06, followed by a slowly decreasing trend from 30 keV to 10 MeV where the ratio changes from 1.06 to 1.02.

Results for AF(BP←BP) and SAF(BP←BP) are shown in figure 11 for the AFB model. For alpha particles, AF ratios at all energies are about 1.0 while the alpha SAF(BP←BP) values using the single-region brain model is 2.0% lower than the SAF(BP←BP) calculated using the dual-region model. This difference in SAF is explained by the 2.1% excess in the mass of the single-region brain model compared to the BOBV region mass in the dual-region brain model.

For low-energy electrons (energies below 100 keV), AF(BP←BP) values using single-region and dual-region brain models are identical and the AF ratio increases up to about 1.02 for 10 MeV electrons. Considering equal AF values for both models, and about 2% excess in the mass of the single-region model compared to the dual-region model, SAF(BP←BP) values at low energy electrons using the single-region model are lower by 2%. For electron energies above 0.1 MeV, the AF(BP←BP) value using the single-region model increases up to a ratio of about 1.02, but this increment is compensated by the excess of mass (about 2%) of the single-region compared to the BOBV region in the dual-region brain model. Consequently, SAF(BP←BP) ratios exhibit the same trend as AF(BP←BP) but with a 2% increment.

For photon sources at very low-energy (about 10 keV), the ratio of AF(BP←BP) using the single-region model to that in the dual-region model increases from 1.01 to 1.02. Above 30 keV, the single-region model shows higher but constant AF(BP←BP) values by ~2%, exactly the mass difference between single and dual-region brain models. For photons above 30 keV, the AF(BP←BP) using the single-region model is about 2% higher than the AF values using the dual-region models. This difference is compensated by the 2% excess in the mass of the single-region models compared to the dual-region models. This compensation results in SAF(BP←BP) ratios of nearly 1.0 above 30 keV photon energies.

AF and SAF approximations for the two target-source combinations (BP←BB and BP←BP) using the AMB single-region and dual-region models are shown in figures 12 and 13, respectively. For all alpha particles and electrons below 100 keV, values of AF(B←B) and SAF(B←B) are shown to be higher by on average 85% and 83% respectively, as compared to AF(BOBV←BB) and SAF(BOBV←BB) values given by the dual-region brain model. Above 30 keV photon energies, AF ratios are ~1.04 and SAF ratios are about 1.02. Below 30 keV, ratios of AF and SAF for photons increase up to 1.35 and 1.33, respectively. For the BP←BP source/target combination, similar tendencies were found in both single-region and dual-region AMB models as compared to those seen in the AFB.

A total of 22 radionuclides with application to radiopharmaceutical therapy, along with 10 radionuclides commonly used in diagnostic imaging were used to compute *S*-values. For the alpha-emitters, *S*-values were also computed for 14 different decay chain progeny corresponding to six different parent alpha-emitters. For both the parent and daughter radionuclides, each reported *S*-value corresponds to an individual radionuclide, and thus branching ratios and biokinetics-derived sums need to be applied to compute a total *S*-value for the entire alpha-emitter decay series. Tables 4 and 5 provide these *S*-values for the adult female brain, while tables 6 and 7 provide them for the adult male brain.

A set of three *S*-values were computed for each radionuclide: S(B←B) from the single-region brain models and both S(BOBV←BB) and S(BOBV←BOBV) from the dual-region brain models. As previously mentioned, values of S(B←B) and S(BOBV←BB) are offered as approximations to the value of S(BP←BB), while values of S(B←B) and S(BOBV←BOBV) are given as approximations to the desired value of S(BP←BP). As mentioned previously, S(BP←BB) and S(BP←BP) represent, respectively, the absorbed dose to brain parenchyma from radionuclide decays in brain blood (defined as a cross-dose component) and from radionuclide decays in the brain parenchyma (defined as a self-dose component).

Ratios of S(B←B) to S(BOBV←BOBV) are displayed in the final column in table 4 for the AFB. These ratios quantify the impact to parenchymal self-dose one can expect in moving from the single-region to the dual-region brain model. Average *S*-value ratios are shown to be 0.982, 0.988, and 0.987 for the alpha-emitters, beta/positron emitters, and Auger electron emitters, respectively, for the therapy-related radionuclides. Thus, this study concludes that using the single-region brain model results in an underestimation of brain parenchymal self-dose of only 1%–2%. In the fourth column of table 4, a similar *S*-value ratio is given between the single-region and dual-region brain models for the cross-dose to brain parenchyma from radionuclide decays within the brain blood. In these cases, the *S*-values ratios are on average 1.922, 1.494, and 1.577, respectively, for the same three categories of therapy-related radionuclides. For these *S*-values, it can be concluded that the conventional single-region brain model overestimates the blood cross-dose to brain parenchyma by between ~50% and 93%. An analogous analysis using the same series of therapy radionuclides for the adult male brain model in table 6 yields average *S*-value ratios of 0.983, 0.988, and 0.987 for brain parenchyma self-dose and average *S*-value ratios of 1.646, 1.370, and 1.419 for blood cross-dose to brain parenchyma.

For the 10 imaging-related radionuclides included in this study, *S*-values ratios for both brain parenchyma self-dose and cross-dose to brain parenchyma from radionuclide decays within the brain blood are shown in the fourth column and the last column of table 5 and table 7, respectively. *S*-value ratios for the cross-dose to brain parenchyma are on average 1.328 and 1.249, ranging from 1.183 to 1.493 and 1.145 and 1.365 for the AFB and AMB, correspondingly. For the parenchyma brain self-dose, average *S*-value ratios of 0.990 and 0.991 were obtained for AFB and AMB models, respectively.

## 5. Conclusions

### 5.1. Significance of this work

SAF values for monoenergetic alpha particles, electrons, positrons, and photons were computed using a dual-region brain model that differentiates between the brain’s main vasculature and the brain parenchyma. The SAF values obtained were compared to SAF computed using the existing single-region brain models of the MRCP adult phantoms from ICRP Publication 145 (ICRP 2020). SAF values considering the brain parenchyma as a target and brain blood as a source given by the single-region brain model were about 2.2 and 1.85 times higher than those from the dual-region brain model, where blood self-dose is explicitly modeled, for both alpha particles (all energies) and low-energy (<100 keV) electrons, in the AFB and AMB correspondingly. In the same way, *S*-values calculated for therapy-related radionuclides using the single-region AFB and AMB models were up to 2.2, 1.9, and 2.1 times higher than those obtained using the dual-region brain models for alpha-emitters, electron/positron emitters, and Auger-electron emitters, respectively. Similarly, calculated *S*-values for SPECT and PET imaging radionuclides using the single-region brain models were up to 49% higher than the *S*-values using the dual-region brain models. The methodology described in this study explicitly allows for the consideration of blood self-dose which is shown to be important for alpha particles (all energies) and electrons at energies below 1.1 MeV.

### 5.2. Model limitations

A limitation of our algorithm is that some vessels intersections were not fixed during the algorithm execution, and thus manual correction was needed as a post-processing step. Our vascular models do not include the pre-capillary and capillary networks of the brain vasculature. Consequently, only a partial volume of brain blood is explicitly modeled by mesh structures in this study. The modeled blood volume represents 47% and 56% of the reference blood volume in the AMB and AFB, respectively. Even though our models do not account for the total blood volume in the brain, the main arteries and veins of radius ranging from several millimeters to 0.18 mm are explicitly modeled for arterial and venous blood circulation. The fact that the minimum vessel radius modeled (0.18 mm) in this study is larger than the range of alpha particles in blood (about 0.164 mm) for the maximum alpha energy (12 MeV) considered is consistent with the fact that the energy deposition of alpha particles created in the BIBV region takes place mostly in the BIBV region. Thus, AF(BOBV←BIBV) is close to zero which causes that the alpha AF(BOBV←BB) to be predominantly defined by the second term in the numerator of equation (6): $\left(1–f_{\text{BV}})\cdot \text{AF}(\text{BOBV}\leftarrow \text{BOBV}\right)$.

As such, the work proposed here provides a significant improvement in estimates of blood self-dose over the existing single-region brain models used in whole-body computational phantoms. Nevertheless, a full accounting of blood self-dose (and the corresponding cross-dose to brain parenchyma would entail a supplemental microscale models of the brain tissues to includes these missing vessel structure. The second limitation of this study is that the developed brain vascular models do not include any type of anastomosis present in real brain vasculature, and rather consider that each brain sub-region has just one entry artery and one entry vein from which the arterial and venous vascular trees are created.

### 5.3. Model applications

The developed AMB and AFB vascular models have two main applications. The first one is for internal dosimetry-applied either to diagnostic imaging or to cancer therapy radionuclides—via a refinement of *S*-values for organ self-dose. With the dual-region brain models, it is now possible to explicitly differentiate decay sites pertinent to radiopharmaceutical tissue localization from those coming from the fraction radiopharmaceutical still in general blood circulation. Our AMB and AFB models allow a transition from a single-region brain model to a dual-region brain model and refine *S* values for blood as a source region. This is particularly important for radionuclides with a short half-life, where dose during wash-in can now be accounted for, or those situations where decay products can re-enter circulation.

The second application of AMB and AFB models is in the field of external beam radiotherapy, where there is increasing interest in dose assessment and sparing of circulating lymphocytes during cancer radiotherapy. Defining the circulating blood cells as an organ-at-risk (OAR), dosimetric techniques are thus essential to compute blood dose-volume histograms (bDVH) to circulating blood cells. Vasculature models such as the one presented here for the brain have been utilized for simulations of bDVH and lymphocytes depletion during external beam radiotherapy as described in our previous work on liver (Xing et al 2022) in combination with compartmental consideration of the remaining whole body (Shin et al 2021).

## Figures and Tables

**Table 1.** PTBV values from a reference study (Hammers et al 2003) and from the segmentation performed in the MRCP AFB/AMB.

			% of TBV in female brain		% of TBV in male brain	
		
	Hammers et al (2003)		AFB		AMB	
		
Brain sub-regions	Median	Std. Dev	Present study	Absolute difference	Present study	Absolute difference

Frontal lobes	31.5	4.5	31.0	−0.50	31.5	0.00
Temporal lobes	21.0	2.9	20.8	−0.20	21.0	0.00
Parietal lobes	20.6	3.1	20.2	−0.40	20.5	−0.10
Occipital lobes	8.2	1.4	8.7	0.50	7.9	−0.30
Cerebellum	10.9	1.5	10.5	−0.40	10.3	−0.60
Brainstem	1.9	0.3	2.1	0.20	2.0	0.10
Central structures	4.5	0.6	4.6	0.10	5.1	0.60
Ventricles	1.3	0.3	1.5	0.20	1.2	−0.10

**Table 2.** Details of the PHITS transport computations and data post-processing for internal dosimetry calculations in the MRCP AFB/AMB.

Item	Description	References

Code and version	PHITS v3.24	Sato et al (2018)
Source description	s-type = 24. Particles are produced uniformly from each tetrahedron thatbelongs to the specified Universe.	Sato et al (2018)
Cross sections	PDL97 for photons EGS5 for photons, electrons and positrons	Cullen et al (1997), Hirayama et al (2005), Boudard et al (2013)
	INCL for nucleons and light ions	
Transport parameters	Secondary electrons were followed for photon simulations. Alpha particles were simulated down to 0.1 MeV/nucleon, while gammas, electrons, and positrons were simulated down to 1 keV.	Sato et al (2018)
Variance reduction	No variance reduction techniques were utilized for this study.	
Statistical uncertainties and history numbers	For the single-region brain model: 1 million photons, electrons, and alpha particles histories were simulated independently at each energy, and relative errors in energy deposition tallies were below 1%.	Sato et al (2018)
	For the dual-region brain model: 1 million photons, electrons, positrons, and alpha particles histories were simulated independently at each energy, and relative errors in energy deposition tallies were below 1% except for 10 keV electrons and positrons and 0.5 MeV alpha particles in which stdcut = 0.01 was incorporated to stop the simulations when the relative errors in energy deposition tallies are below 1% in a batch.	
Data and post-processing	Energy deposited (MeV/source) was tallied in the single-region brain models. Absorbed fractions were calculated by normalizing the results to the particle source energy. Energy deposited (MeV/source) was tallied in the BOBV region of the dualregion brain model. Absorbed fractions from the following source-target combinations: (BOBV <- BOBV) and (BOBV <- BIBV) were calculated by normalizing the results to the particle source energy at each target. The fraction of blood mass was used to weight average the absorbed fractions (See equation (6)).	Correa-Alfonso et al (2022)

**Table 3.** Tissue elemental compositions, mass densities, and total tissue masses assigned in the PHITS transport models for internal dosimetry calculations in the MRCP AFB and AMB (ICRU 1992).

Tissue component	Elemental composition (% by mass)												
	H	C	N	O	Na	P	S	Cl	K	Fe	Density (g cm^3^)	Source	

Adult healthy brain	10.70	14.50	2.20	71.20	0.20	0.40	0.20	0.30	0.30	0.00	1.04	ICRU 46	
Adult whole blood	10.20	11.00	3.30	74.50	0.10	0.10	0.20	0.30	0.20	0.10	1.06	ICRU 46	
Adult female brain (AFB)													Mass (kg)
Homogeneous brain (B)	10.68	14.37	2.24	71.32	0.20	0.39	0.20	0.30	0.30	0.00	1.0407		1.350
Brain inside BV (BIBV)	10.20	11.00	3.30	74.50	0.10	0.10	0.20	0.30	0.20	0.10	1.060	ICRU 46	0.028
Brain outside BV (BOBV)	10.69	14.44	2.22	71.25	0.20	0.40	0.20	0.30	0.30	0.00	1.0405		1.322
Adult male brain (AMB)													
Homogeneous liver (L)	10.68	14.34	2.25	71.35	0.20	0.39	0.20	0.30	0.30	0.00	1.0409		1.517
Brain inside BV (BIBV)	10.20	11.00	3.30	74.50	0.10	0.10	0.20	0.30	0.20	0.10	1.060	ICRU 46	0.032
Brain outside BV (BOBV)	10.69	14.42	2.23	71.28	0.20	0.39	0.20	0.30	0.30	0.00	1.0403		1.486

**Table 4.** Approximations of S(BP ← BB) and S(BP ← BP) values for 22 radionuclides (and 14 additional alpha-emitter decay progeny) with applications to radiopharmaceutical therapy using the single-region and dual-regiontetrahedral mesh models of the reference adult female brain (AFB).

Radionuclide	*S*-values (mGy/MBq-s)					Ratio
	Approximations to S(BP ← BB)		Ratio	Approximations to S(BP ← BP)		
	S(B ← B)	S(BOBV ← BB)		S(B ← B)	S(BOBV ← BOBV)	

Alpha Emitters^a^						
At-211	2.99E-04	1.36E-04	2.189	2.99E-04	3.05E-04	0.979
(Po-211)	9.00E-04	4.13E-04	2.178	9.00E-04	9.19E-04	0.979
(Bi-207)	4.19E-05	3.66E-05	1.145	4.19E-05	4.22E-05	0.994
Bi-212	3.23E-04	1.65E-04	1.961	3.23E-04	3.29E-04	0.981
(Po-212)	1.06E-03	4.92E-04	2.161	1.06E-03	1.08E-03	0.979
(Tl-208)	1.19E-04	9.55E-05	1.244	1.19E-04	1.20E-04	0.992
Bi-213	6.82E-05	4.27E-05	1.597	6.82E-05	6.92E-05	0.986
(Po-213)	1.01E-03	4.68E-04	2.166	1.01E-03	1.03E-03	0.979
(Tl-209)	1.15E-04	9.10E-05	1.269	1.15E-04	1.16E-04	0.991
(Pb-209)	2.33E-05	1.29E-05	1.806	2.33E-05	2.37E-05	0.983
Ra-223	6.97E-04	3.19E-04	2.187	6.97E-04	7.12E-04	0.979
(Rn-219)	8.18E-04	3.75E-04	2.183	8.18E-04	8.36E-04	0.979
(Po-215)	8.93E-04	4.10E-04	2.179	8.93E-04	9.12E-04	0.979
(Pb-211)	5.41E-05	3.63E-05	1.490	5.41E-05	5.48E-05	0.987
(Bi-211)	7.94E-04	3.63E-04	2.186	7.94E-04	8.11E-04	0.979
(Tl-207)	5.76E-05	3.89E-05	1.480	5.76E-05	5.84E-05	0.987
(Po-211)	9.00E-04	4.13E-04	2.178	9.00E-04	9.19E-04	0.979
Ac-225	7.02E-04	3.20E-04	2.196	7.02E-04	7.17E-04	0.979
(Fr-221)	7.63E-04	3.49E-04	2.190	7.63E-04	7.80E-04	0.979
(At-217)	8.54E-04	3.91E-04	2.183	8.54E-04	8.73E-04	0.979
Th-227	7.22E-04	3.31E-04	2.185	7.22E-04	7.38E-04	0.979
Beta and positron emitters						
Sr-89	6.77E-05	4.74E-05	1.430	6.77E-05	6.85E-05	0.988
Y-90	1.07E-04	8.23E-05	1.294	1.07E-04	1.08E-04	0.991
I-124	4.28E-05	3.59E-05	1.191	4.28E-05	4.31E-05	0.993
I-131	3.02E-05	1.95E-05	1.544	3.02E-05	3.06E-05	0.986
Sm-153	3.39E-05	1.94E-05	1.747	3.39E-05	3.44E-05	0.984
Ho-166	8.14E-05	5.81E-05	1.402	8.14E-05	8.24E-05	0.989
Lu-177	1.83E-05	9.74E-06	1.875	1.83E-05	1.86E-05	0.982
Re-186	3.99E-05	2.48E-05	1.610	3.99E-05	4.05E-05	0.985
Re-188	9.11E-05	6.74E-05	1.351	9.11E-05	9.20E-05	0.990
Auger electron emitters						
Pd-103	1.98E-06	1.51E-06	1.308	1.98E-06	2.00E-06	0.992
In-111	1.34E-05	1.09E-05	1.232	1.34E-05	1.35E-05	0.992
Sn-117m	2.31E-05	1.31E-05	1.769	2.31E-05	2.35E-05	0.983
I-123	7.95E-06	5.96E-06	1.334	7.95E-06	8.03E-06	0.990
I-125	5.12E-06	3.69E-06	1.387	5.12E-06	5.18E-06	0.990
Pt-193m	1.69E-05	8.19E-06	2.060	1.69E-05	1.72E-05	0.980
Pt-195m	2.46E-05	1.26E-05	1.948	2.46E-05	2.50E-05	0.981

a Radionuclides in parentheses for alpha emitters such as (Po-211) correspond to alpha-emitter decay progeny.

**Table 5.** Approximations of S(BP ← BB) and S(BP ← BP) values for 10 radionuclides with applications to diagnostic imaging using the single-region and dual-region tetrahedral mesh models of the reference adult female brain (AFB).

Radionuclide	*S*-values (mGy/MBq-s)					Ratio
	Approximations to S(BP ← BB)		Ratio	Approximations to S(BP ← BP)		
	S(B ← B)	S(BOBV ← BB)		S(B ← B)	S(BOBV ← BOBV)	

SPECT radionuclides						
Ga-67	8.01E-06	5.36E-06	1.493	8.01E-06	8.11E-06	0.987
Tc-99m	4.57E-06	3.43E-06	1.331	4.57E-06	4.61E-06	0.990
In-111	1.34E-05	1.09E-05	1.232	1.34E-05	1.35E-05	0.992
I-123	7.95E-06	5.96E-06	1.334	7.95E-06	8.03E-06	0.990
PET radionuclides						
C-11	6.46E-05	4.68E-05	1.380	6.46E-05	6.53E-05	0.989
N-13	7.69E-05	5.66E-05	1.359	7.69E-05	7.78E-05	0.989
O-15	1.04E-04	8.03E-05	1.302	1.04E-04	1.05E-04	0.991
F-18	4.75E-05	3.42E-05	1.388	4.75E-05	4.80E-05	0.989
Ga-68	1.03E-04	8.06E-05	1.282	1.03E-04	1.04E-04	0.991
Rb-82	1.79E-04	1.52E-04	1.183	1.79E-04	1.80E-04	0.994

**Table 6.** Approximations of S(BP ← BB) and S(BP ← BP) values for 22 radionuclides (and 14 additional alpha-emitter decay progeny) with applications to radiopharmaceutical therapy using the single-region and dual-region tetrahedral mesh models of the reference adult male brain (AMB).

Radionuclide	*S*-values (mGy/MBq-s)					
	Approximations to S(BP ← BB)		Ratio	Approximations to S(BP ← BP)		Ratio
	S(B ← B)	S(BOBV ← BB)		S(B ← B)	S(BOBV ← BOBV)	

Alpha emitters^a^						
At-211	2.66E-04	1.46E-04	1.820	2.66E-04	2.71E-04	0.979
(Po-211)	8.01E-04	4.42E-04	1.814	8.01E-04	8.18E-04	0.980
(Bi-207)	3.81E-05	3.44E-05	1.108	3.81E-05	3.83E-05	0.995
Bi-212	2.88E-04	1.71E-04	1.684	2.88E-04	2.93E-04	0.981
(Po-212)	9.45E-04	5.24E-04	1.804	9.45E-04	9.65E-04	0.980
(Tl-208)	1.07E-04	9.05E-05	1.187	1.07E-04	1.08E-04	0.992
Bi-213	6.08E-05	4.20E-05	1.447	6.08E-05	6.16E-05	0.986
(Po-213)	9.01E-04	4.99E-04	1.807	9.01E-04	9.20E-04	0.980
(Tl-209)	1.04E-04	8.61E-05	1.207	1.04E-04	1.05E-04	0.992
(Pb-209)	2.07E-05	1.30E-05	1.588	2.07E-05	2.10E-05	0.983
Ra-223	6.20E-04	3.41E-04	1.818	6.20E-04	6.33E-04	0.979
(Rn-219)	7.28E-04	4.01E-04	1.817	7.28E-04	7.43E-04	0.979
(Po-215)	7.94E-04	4.38E-04	1.814	7.94E-04	8.11E-04	0.980
(Pb-211)	4.82E-05	3.51E-05	1.373	4.82E-05	4.88E-05	0.988
(Bi-211)	7.06E-04	3.88E-04	1.818	7.06E-04	7.21E-04	0.979
(Tl-207)	5.13E-05	3.75E-05	1.367	5.13E-05	5.19E-05	0.988
(Po-211)	8.01E-04	4.42E-04	1.814	8.01E-04	8.18E-04	0.980
Ac-225	6.25E-04	3.43E-04	1.824	6.25E-04	6.38E-04	0.979
(Fr-221)	6.79E-04	3.73E-04	1.820	6.79E-04	6.94E-04	0.979
(At-217)	7.60E-04	4.18E-04	1.817	7.60E-04	7.76E-04	0.979
Th-227	6.43E-04	3.54E-04	1.818	6.43E-04	6.56E-04	0.979
Beta and positron emitters						
Sr-89	6.03E-05	4.53E-05	1.331	6.03E-05	6.10E-05	0.989
Y-90	9.50E-05	7.71E-05	1.232	9.50E-05	9.58E-05	0.992
I-124	3.89E-05	3.39E-05	1.147	3.89E-05	3.91E-05	0.994
I-131	2.71E-05	1.93E-05	1.404	2.71E-05	2.75E-05	0.987
Sm-153	3.02E-05	1.95E-05	1.546	3.02E-05	3.07E-05	0.984
Ho-166	7.25E-05	5.53E-05	1.312	7.25E-05	7.33E-05	0.989
Lu-177	1.63E-05	9.98E-06	1.630	1.63E-05	1.66E-05	0.982
Re-186	3.55E-05	2.44E-05	1.457	3.55E-05	3.60E-05	0.986
Re-188	8.12E-05	6.37E-05	1.274	8.12E-05	8.20E-05	0.990
Auger electron emitters						
Pd-103	1.87E-06	1.49E-06	1.253	1.87E-06	1.88E-06	0.991
In-111	1.23E-05	1.05E-05	1.177	1.23E-05	1.24E-05	0.993
Sn-117m	2.09E-05	1.34E-05	1.554	2.09E-05	2.12E-05	0.984
I-123	7.41E-06	5.94E-06	1.248	7.41E-06	7.48E-06	0.990
I-125	4.95E-06	3.86E-06	1.282	4.95E-06	5.00E-06	0.989
Pt-193m	1.50E-05	8.60E-06	1.745	1.50E-05	1.53E-05	0.980
Pt-195m	2.18E-05	1.30E-05	1.676	2.18E-05	2.22E-05	0.981

a Radionuclides in parentheses for alpha emitters such as (Po-211) correspond to alpha-emitter decay progeny

**Table 7.** Approximations of S(BP ← BB) and S(BP ← BP) values for 10 radionuclides with applications to diagnostic imaging using the single-region and dual-region tetrahedral mesh models of the reference adult male brain (AMB).

Radionuclide	*S*-values (mGy/MBq-s)					
	Approximations to S(BP ← BB)		Ratio	Approximations to S(BP ← BP)		Ratio
	S(B ← B)	S(BOBV ← BB)		S(B ← B)	S(BOBV ← BOBV)	

SPECT radionuclides						
Ga-67	7.22E-06	5.29E-06	1.365	7.22E-06	7.32E-06	0.987
Tc-99m	4.16E-06	3.34E-06	1.247	4.16E-06	4.20E-06	0.990
In-111	1.23E-05	1.05E-05	1.177	1.23E-05	1.24E-05	0.993
I-123	7.41E-06	5.94E-06	1.248	7.41E-06	7.48E-06	0.990
PET radionuclides						
C-11	5.81E-05	4.51E-05	1.288	5.81E-05	5.88E-05	0.989
N-13	6.91E-05	5.43E-05	1.274	6.91E-05	6.98E-05	0.990
O-15	9.37E-05	7.59E-05	1.234	9.37E-05	9.45E-05	0.991
F-18	4.29E-05	3.32E-05	1.292	4.29E-05	4.33E-05	0.989
Ga-68	9.26E-05	7.59E-05	1.220	9.26E-05	9.34E-05	0.992
Rb-82	1.61E-04	1.40E-04	1.145	1.61E-04	1.62E-04	0.994

## Data Availability

All data that support the findings of this study are included within the article (and any supplementary information files). Data will be available from 1 March 2023.

## Footnotes

4 Robert McNeel & Associates, Seattle, WA.

5 ANSYS, Inc. San Jose CA.

6 Robert McNeel & Associates, Seattle, WA.

7 A bad mesh refers to a mesh that has self-intersections, non-manifold edges, duplicated faces or degenerated faces.

8 TETView http://tetgen.berlios.de/tetview. html. 2020, v2.

8 http://www.nist.gov/.
